# Characterization and Mitigation of a Simultaneous Multi‐Slice fMRI Artifact: Multiband Artifact Regression in Simultaneous Slices

**DOI:** 10.1002/hbm.70066

**Published:** 2024-11-06

**Authors:** Philip N. Tubiolo, John C. Williams, Jared X. Van Snellenberg

**Affiliations:** ^1^ Department of Biomedical Engineering Stony Brook University Stony Brook New York USA; ^2^ Department of Psychiatry and Behavioral Health Renaissance School of Medicine at Stony Brook University Stony Brook New York USA; ^3^ Department of Psychology Stony Brook University Stony Brook New York USA

**Keywords:** artifact, denoising, fMRI, multiband, simultaneous multi‐slice, task‐based fMRI, working memory

## Abstract

Simultaneous multi‐slice (multiband) acceleration in fMRI has become widespread, but may be affected by novel forms of signal artifact. Here, we demonstrate a previously unreported artifact manifesting as a shared signal between simultaneously acquired slices in all resting‐state and task‐based multiband fMRI datasets we investigated, including publicly available consortium data from the Human Connectome Project (HCP) and Adolescent Brain Cognitive Development (ABCD) Study. We propose Multiband Artifact Regression in Simultaneous Slices (MARSS), a regression‐based detection and correction technique that successfully mitigates this shared signal in unprocessed data. We demonstrate that the signal isolated by MARSS correction is likely nonneural, appearing stronger in neurovasculature than gray matter. Additionally, we evaluate MARSS both against and in tandem with sICA+FIX denoising, which is implemented in HCP resting‐state data, to show that MARSS mitigates residual artifact signal that is not modeled by sICA+FIX. MARSS correction leads to study‐wide increases in signal‐to‐noise ratio, decreases in cortical coefficient of variation, and mitigation of systematic artefactual spatial patterns in participant‐level task betas. Finally, MARSS correction has substantive effects on second‐level *t*‐statistics in analyses of task‐evoked activation. We recommend that investigators apply MARSS to multiband fMRI datasets with moderate or higher acceleration factors, in combination with established denoising methods.

## Graphical abstract

We present a previously unreported artifact signal shared between simultaneously acquired slices in resting‐state and task‐based multiband fMRI. We developed Multiband Artifact Regression in Simultaneous Slices (MARSS) to estimate and remove this artifact, leading to improved data quality and substantive changes to task‐based fMRI analysis results within and across participants.

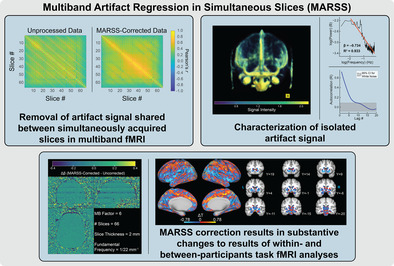

**Summary**

- We present a previously unreported artifact signal shared between simultaneously acquired slices in multiband fMRI that is observed in multiple task‐based and resting‐state datasets, including in publicly available consortium data.
- We propose Multiband Artifact Regression in Simultaneous Slices (MARSS), a regression‐based method that estimates and removes this artifact signal in unprocessed multiband fMRI data.
- MARSS correction leads to widespread increases in temporal signal‐to‐noise ratio, decreases in cortical coefficient of variation, and substantive changes to both within‐participant estimates of task‐evoked activation and between‐participant spatial *t*‐statistics.

## 1 Introduction

### 1.1 Principles and Advantages of Multiband fMRI

Simultaneous multi‐slice (multiband; MB) functional magnetic resonance imaging (fMRI) has become a prevalent fMRI acquisition acceleration technique that partially mitigates the limitations posed by standard single‐slice (single‐band) EPI acquisition (Feinberg et al. 2010; Moeller et al. 2010; Xu et al. 2013). In MB fMRI acquisition, composite radiofrequency pulses comprising central frequencies (with bandwidths that determine slice thickness) that match the precession frequency of hydrogen nuclei in several axial sections along the field of view are transmitted to acquire signal from several slices in a single readout; the number of slices acquired simultaneously is denoted by the MB acceleration factor. During readout, simultaneously acquired slices are “stacked” on top of each other and phase‐shifted along the phase‐encoding direction to reduce the amount of overlapping signal between slices (Breuer et al. 2005; Setsompop et al. 2011). Slices are then unmixed using matrix inversion techniques that utilize radiofrequency coil sensitivity maps acquired prior to image acquisition. Overall, MB acceleration allows for substantive increases in spatiotemporal resolution that reduce signal‐to‐noise ratio but improve the spatial precision of neural signal estimates (Feinberg et al. 2010). These improvements in data quality have led to its widespread adoption in both individual research laboratories and in large consortiums that have produced publicly available datasets, such as the Human Connectome Project (Van Essen et al. 2013), UK Biobank study (Miller et al. 2016), and Adolescent Brain Cognitive Development (ABCD) Study (Casey et al. 2018; Jernigan, Brown, and Dowling 2018).

### 1.2 Previously Discovered Multiband‐Specific Artifacts and Mitigation Methods

As MB acceleration has gained adoption, several sources of contamination stemming from MB acquisition have been identified. Todd et al. (2016) identified a source of shared signal among voxels in simultaneously acquired slices that were overlapped during readout, coined “interslice leakage.” It was determined that this artifact could be substantially reduced by using split‐slice generalized autocalibrating partial parallel acquisition (GRAPPA; Cauley et al. 2014) image reconstruction over other techniques, such as Slice‐GRAPPA (Griswold et al. 2002). McNabb and associates subsequently identified that interslice leakage artifacts are exacerbated by eye movement, even when Split‐splice GRAPPA reconstruction is used, by having participants forcefully blink during resting‐state MB fMRI acquisition (McNabb et al. 2020).

Additionally, MB fMRI acquisition poses unique challenges in the estimation and removal of artifacts due to in‐scanner participant motion, as the higher sampling rate enabled by these sequences facilitates the capture of additional nuisance signals (Burgess et al. 2016; Fair et al. 2020; Glasser et al. 2018; Power et al. 2019; Williams et al. 2022), including respiratory motion and pseudomotion, or factitious head motion observed in the phase‐encode direction as a consequence of lung expansion distorting the *B*_0_ field (Fair et al. 2020; Power et al. 2019).

### 1.3 Discovery of a Shared Artifact Signal in Simultaneously Acquired Slices

**FIGURE 1:**
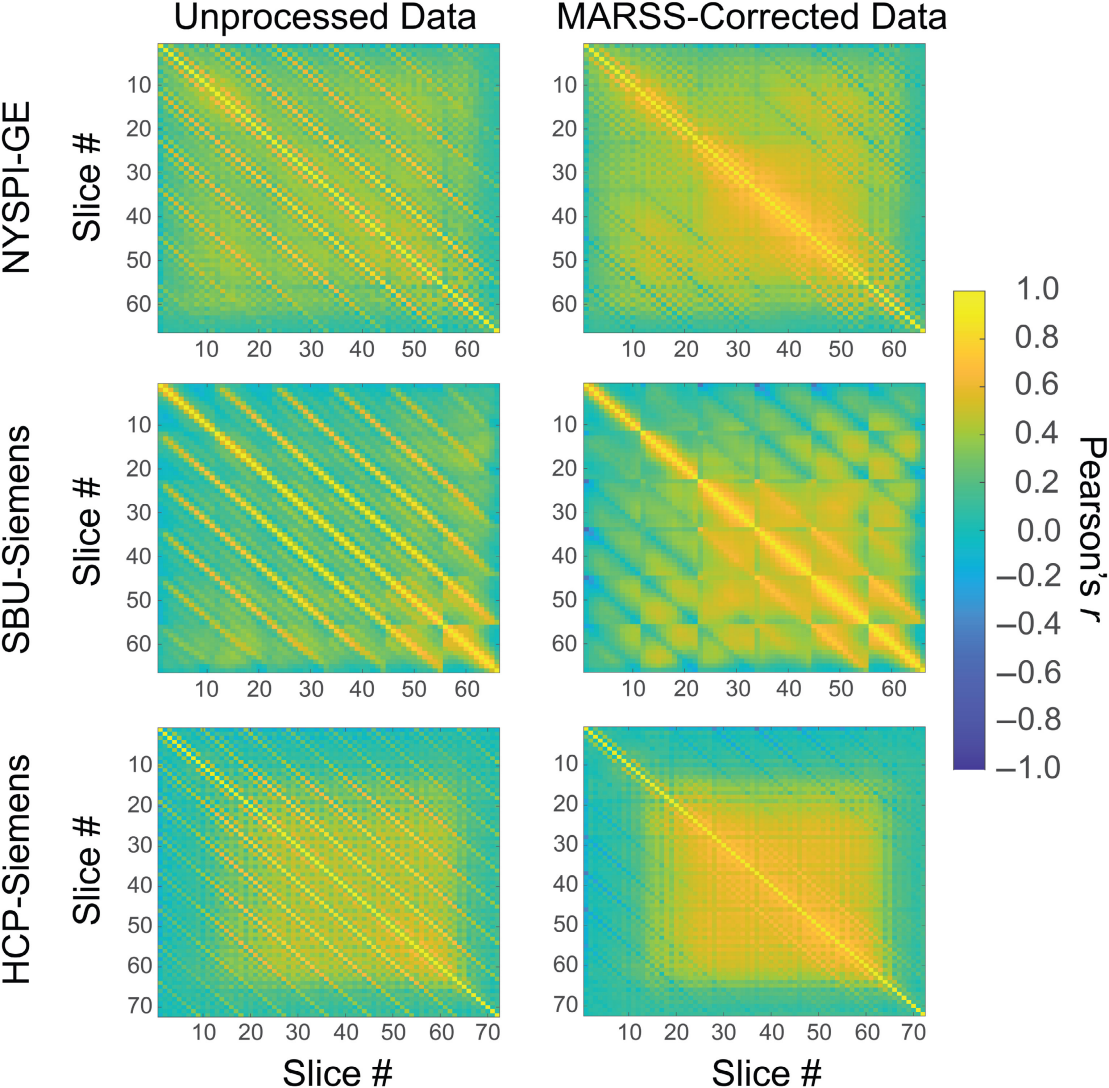
Pearson correlation matrices between average signal in all slice pairs in unprocessed and MARSS‐corrected task‐based data from NYSPI‐GE (*N* = 58), SBU‐Siemens (*N* = 10), and HCP‐Siemens (*N* = 25).

In working with resting‐state and task‐based MB fMRI data in our own laboratory, we observed periodic banding patterns in carpet plots (Power et al. 2014) that display mean slice‐wise timeseries. In investigating the source of this banding pattern, we discovered that raw, unprocessed MB fMRI data display elevated Pearson correlations between the average timeseries in simultaneously acquired slices, visible as diagonal bands in a correlation matrix of average slices signals in the original scanner space (Figure 1). Given that there is no biological basis for true neural signal fluctuations to be shared between arbitrarily located, spatially disparate, axial slices over and above that which is shared with other slices in the image, we hypothesized that these elevated correlations represent a multiband fMRI signal artifact: a nonneural, structured signal source that is shared across simultaneously acquired slices and uniquely detectable in MB accelerated fMRI. Notably, our use of the term “artifact” to refer to this signal may differ somewhat from some definitions of an “image artifact” or “multiband artifact.” We do not mean to assert that this artifact is somehow produced or created by the image sequence or multiband implementation, although this remains a possibility. Rather, we use the term “multiband artifact” to describe an undesirable, nonneural, signal source that negatively impacts data quality and that is present in BOLD multiband imaging. Indeed, this signal source could still exist in single‐band fMRI, but if so, it would be undetectable because no two slices are acquired simultaneously.

Consequently, we detail the identification of this novel artifact signal across several resting‐state and task‐based MB fMRI datasets, including data from two large, publicly available, datasets (Casey et al. 2018; Jernigan, Brown, and Coordinators 2018; Van Essen et al. 2013), and three major scanner manufacturers: Siemens (Munich, Germany), General Electric (GE; Boston, MA), and Philips (Andover, MA). We propose a regression‐based technique to reduce the presence of the shared signal between simultaneously acquired slices, via estimation and removal of the shared signal in unprocessed MB fMRI data files, which we term Multiband Artifact Regression in Simultaneous Slices (MARSS). This estimation method is based on the idea that, provided major sources of shared signal (i.e., global signal and motion‐related signal) are separately accounted for, averaging together a sufficient number of voxels containing the same artifact signal (i.e., all voxels from all simultaneously acquired slices) will converge on the artifact signal itself, as true neural signals from disparate brain regions will “average out.”

Furthermore, we determine whether this artifact signal is distinct from previously identified interslice leakage artifacts. We also perform a preliminary investigation of the spatial, temporal, and spectral properties of the isolated artifact signal. We then explore whether denoising via both spatial independent components analysis with FMRIB's ICA‐based Xnoiseifier (sICA+FIX; Griffanti et al. 2014; Salimi‐Khorshidi et al. 2014) and retrospective correction of physiological motion effects (RETROICOR; Glover, Li, and Ress 2000) mitigates this artifact signal when used in isolation and in tandem with MARSS. Finally, we perform a working memory task analysis in both processed and unprocessed MB fMRI to highlight the effects of MARSS correction on spatial patterns of estimates of task‐evoked activation. We predicted that the artifact signal may originate from both the transit of perturbed proton spins in a periodic fashion through fluid‐filled spaces in the brain and periodic mechanical noise during acquisition. Further, we hypothesized that noise reduction due to artifact removal would result in increased magnitude of spatial *t*‐statistics in between‐participants analyses of task‐based fMRI, most likely as a result of reduced variance due to removal of spurious artifact signals.

## 2 Materials and Methods

### 2.1 Functional Magnetic Resonance Imaging Datasets

To comprehensively evaluate the presence of the artifact signal across a range of scanner platforms and scan parameters, we utilized phantom and in vivo MB fMRI data from several sources, including samples from two publicly available datasets (HCP and ABCD), and two in‐house samples acquired at the New York State Psychiatric Institute (NYSPI) and Stony Brook University (SBU). A comprehensive data summary including scan acquisition parameters can be found in Table 1, and demographic information for each dataset in Table S1.

**TABLE 1:** Datasets used in this study.

Dataset ID	Scanner(s)	# Subjects	Phantom/In Vivo	MB	TR (ms)	TE (ms)	FOV (mm)	Matrix Size	Flip Angle (°)	Partial Fourier Factor	Voxel Size	Task(s)
NYSPI‐GE	GE MR 750 3 T	*N* = 66	In Vivo	6	850	25	204 × 204	102 × 102	60	17/24	2 mm isotropic	RS (*N* = 63), SOT (*N* = 58)
		*N* = 1	In Vivo	3	1325	25	204 × 204	102 × 102	60	17/24	2 mm isotropic	RS
				5	850		204 × 204	102 × 102				
				7	800		192 × 192	96 × 96				
		*N* = 1	Phantom	4	1300	25	204 × 204	102 × 102	60	17/24	2 mm isotropic	RS
				6	850		204 × 204	102 × 102				
				8	850		192 × 192	96 × 96				
SBU‐Siemens	Siemens Prisma 3 T	*N* = 10	In Vivo	6	800	25	204 × 204	102 × 102	60	6/8	2 mm isotropic	RS (*N* = 10), SOT (*N* = 10)
		*N* = 1	In Vivo	3	1580	25	204 × 204	102 × 102	60	6/8	2 mm isotropic	RS
				5	939							
				7	657							
		*N* = 1	Phantom	4	1505	25	204 × 204	102 × 102	60	6/8	2 mm isotropic	RS
				6	1010							
				8	798							
ABCD‐Siemens	Siemens Prisma 3 T; Prisma Fit 3 T	*N* = 25 (Prisma *N* = 8; Prisma Fit *N* = 17)	In Vivo	6	800	30	216 × 216	90 × 90	52	N/A	2.4 mm isotropic	RS
ABCD‐GE	GE MR750 3 T	*N* = 3	In Vivo	6	800	30	216 × 216	90 × 90	52	N/A	2.4 mm isotropic	RS
ABCD‐Philips	Philips Achieva dStream 3 T; Ingenia 3 T	*N* = 7 (dStream *N* = 5; Ingenia *N* = 2)	In Vivo	6	800	30	216 × 216	90 × 90	52	N/A	2.4 mm isotropic	RS
HCP‐Siemens	Siemens Connectome Skyra 3 T	*N* = 25	In Vivo	8	720	33.1	208 × 180	104 × 90	52	N/A	2 mm isotropic	RS (*N* = 25), WM (*N* = 25)

*Note:* Datasets are referred to by their Dataset ID throughout the manuscript.

Abbreviations: ABCD = Adolescent Brain Cognitive Development; FOV = Field Of View; HCP = Human Connectome Project; MB = multiband acceleration factor; NYSPI = New York State Psychiatric Institute; RS = resting‐state; SBU = Stony Brook University; SOT = self‐ordered working memory task; TE = echo time; TR = repetition time; WM = working memory task.

All procedures described in this study that involve the acquisition, curation, and analysis of data from human participants at NYSPI and SBU were approved by the Institutional Review Boards of NYSPI—Columbia University Department of Psychiatry and SBU, respectively. All participants provided written consent prior to their participation in study procedures.

#### 2.1.1 Resting‐State In Vivo Datasets

We first employed unprocessed resting‐state MB fMRI data acquired at NYSPI (NYSPI‐GE; *n* = 63), with between one and four runs per participant (one participant had one run, four had two runs, nine had three runs, and 49 had four runs). Another resting‐state dataset was acquired at SBU (SBU‐Siemens; *n* = 10), with four or six runs per participant (one participant had six runs). NYSPI‐GE data were collected using a Nova Medical (Wilmington, MA) 32‐channel head coil, while SBU‐Siemens data used a Siemens 64‐channel head‐and‐neck coil.

We also used resting‐state data from a subset of the HCP (HCP‐Siemens; Smith et al. 2013; Ugurbil et al. 2013; Van Essen et al. 2013) 500 subjects release (*n* = 25) and the ABCD Study (ABCD‐Siemens, ABCD‐GE, and ABCD‐Philips; *n* = 35; Casey et al. 2018; Jernigan, Brown, and Coordinators 2018; Jernigan, Brown, and Dowling 2018; Volkow et al. 2018). All participants in the HCP‐Siemens dataset had four runs of resting‐state data, while participants in the ABCD datasets had a minimum of one and maximum of five runs of resting‐state data.

To evaluate the relationship between artifact presence and MB factor in vivo, one participant each from SBU‐Siemens and NYSPI‐GE underwent one resting‐state run for each MB factor of 3, 5, and 7.

Notably, all MB fMRI data collected at SBU were reconstructed using split‐slice GRAPPA (Cauley et al. 2014), and all data acquired at NYSPI and through the HCP were reconstructed using blipped Controlled Aliasing in Parallel Imaging Results in Higher Acceleration (CAIPIRINHA; Setsompop et al. 2011).

#### 2.1.2 Task‐Based Datasets

Up to four runs of task‐based MB fMRI data were acquired from participants in the SBU‐Siemens dataset (*n* = 10; one participant had three runs) and a subset of the NYSPI‐GE dataset (*n* = 58; eight participants had one run, four had two runs, 14 had three runs, and 32 had four runs) using identical acquisition parameters to the resting‐state data. Participants performed the self‐ordered working memory task (SOT; described in the Task Procedures section of Data S1; Van Snellenberg et al. 2015). Additionally, each HCP‐Siemens participant (*n* = 25) performed two runs of working memory task‐based data (Barch et al. 2013; Ugurbil et al. 2013; Van Essen et al. 2013) with identical acquisition parameters to HCP‐Siemens resting‐state data.

#### 2.1.3 Phantom Datasets

Phantom data were acquired to investigate both the nonneural origin of the artifact signal and the relationship between artifact presence and MB factor. Three runs of a Siemens D165 Spherical Phantom containing a Nickel sulfate solution were acquired at SBU, and three runs of an agar‐filled Functional Biomedical Informatics Research Network (FBIRN) spherical stability phantom (Friedman and Glover 2006) were acquired at NYSPI.

#### 2.1.4 Single‐Band Datasets

We utilized two single‐band fMRI datasets (see Table S2) in order to compare slice‐wise correlation patterns in MB fMRI data to those obtained without the use of MB acceleration. The first dataset comprised 27 participants collected at NYSPI (NYSPI‐Philips; Girgis et al. 2016; Van Snellenberg et al. 2016; Williams et al. 2023) completing the SOT (*N* = 27; Van Snellenberg et al. 2016) and an *n*‐back working memory task (*N* = 22; Williams et al. 2023). The second dataset comprised publicly available data from 46 participants in the Queensland Twin Imaging Study (QTIM‐Bruker; Blokland et al. 2011; Sinclair et al. 2015) undergoing one run of resting‐state fMRI and one run of an *n*‐back working memory task.

### 2.2 Identification of Artifact Signal in Simultaneously Acquired Slices

A shared artifact signal across simultaneously acquired slices was identified separately in each run of MB fMRI data across all datasets. For each run, the average timeseries in each axial slice was obtained. To remove the impact of participant head motion on raw slice timeseries data, each slice timeseries was then taken to be the residuals of a multiple linear regression modeling the average slice timeseries as a function of six rigid‐body, volume‐wise head motion parameters obtained via SPM12 (Friston 2004, 2007), their squares, derivatives, and squared derivatives. For each run, Pearson correlations were calculated between all pairs of slice‐wise timeseries in the image. The presence of a shared artifact signal across simultaneously acquired slices was quantified as the difference in the mean Pearson correlation between the timeseries in simultaneously acquired slices and the mean correlation between the pairs of slices immediately above and below each slice in the simultaneous slice set along the z‐direction (herein simply referred to as “adjacent‐to‐simultaneous” slices), averaged across all slices in a volume.

First, given a run with $N_{s}$ slices and a MB acceleration factor ${\mathrm{MB}}_{f}$, the number of simultaneously acquired slices is given by:

(1) $$N_{\mathrm{MB}}=\frac{N_{s}}{{\mathrm{MB}}_{f}}$$

Given a slice j from this run, we define the set of indices of slices acquired simultaneous to j as:

(2) $$\begin{array}{c} U_{j}=\left\{u|\;u=m\cdot c_{j}\wedge u\leq N_{s}\right\}\; \end{array}$$

where $N_{s}$ is the total number of slices in the volume, $\;m\in {\mathbb{N}}^{+}$, and:
(3)

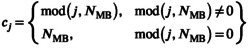




Now the set of indices of adjacent‐to‐simultaneous slices associated with set $U_{j}$ can be defined as:

(4) $$\Upsilon_{j}=\left\{y|y=x\pm 1,x\in U_{j},1\leq x\leq N_{s}\right\}$$

As shown, each element in $\Upsilon_{j}$ is bound between one and the number of slices; therefore, slices for which $U_{j}$ includes either the first or last slice of each image will have one fewer adjacent‐to‐simultaneous slice than sets that do not, as the first and last slices only have one slice adjacent to them. Adjacent‐to‐simultaneous slices were chosen as a comparison because these slices are assumed to share anatomical and functional similarity to the given slice in the simultaneous slice set, as well as the intrinsic smoothness of BOLD data. Thus, the null expectation (assuming no multiband artifact signal) is that correlations between simultaneously acquired slices should be no higher than the correlations between adjacent‐to‐simultaneous slices. Correlations between slice‐wise timeseries in $U_{j}$ for each slice j were averaged together before being averaged across all simultaneous slice sets. The same averaging was performed for $\Upsilon_{j}$, and resulting mean correlations were then subtracted. If we define $X_{j}$ as the mean signal in slice j, then

(5) $$r_{U‐\Upsilon }=\sum_{j=1}^{N_{s}}\sum_{k\in U_{j}}\text{artanh}\left(\text{corr}\left(X_{j},X_{k}\right)\right)/\left(N_{s}\times \left|U_{j}\right|\right)-\sum_{j=1}^{N_{s}}\sum_{k\in \Upsilon_{j}}\text{artanh}\left(\text{corr}\left(X_{j},X_{k}\right)\right)/\left(N_{s}\times \left|\Upsilon_{j}\right|\right)$$

where artanh is the inverse hyperbolic tangent, also known in this context as Fisher's *r*‐to‐*Z* transformation (note that all Pearson correlations were Fisher *Z*‐transformed prior to averaging or subtracting and transformed back to Pearson *r* before being reported throughout this manuscript). This procedure was performed before and after artifact correction techniques were used to quantify the magnitude of artifact and success of artifact removal. Statistical significance of elevated correlations between simultaneously acquired slices in uncorrected data and the reduction in these correlations in MARSS‐corrected data were determined via Wilcoxon signed‐rank tests (Gibbons and Chakraborti 2021; Hollander, Wolfe, and Chicken 2014).

### 2.3 Multiband Artifact Regression in Simultaneous Slices

#### 2.3.1 MARSS Estimation of Artifact Signal

We developed a regression‐based method, MARSS, to estimate the magnitude of, and remove, the artifact signal shared across simultaneously acquired slices for each run of MB fMRI data. Using the definitions above in Equations ((1), (2), (3)), we define $d_{i,u}$ as the timeseries in voxel i of slice u, which is a slice in $U_{j}$, the set of slices acquired simultaneously to slice j. The average of timeseries across all $d_{i,u}$ for all slices in $U_{j}$, but excluding slice j, is defined as:

(6) $$\begin{array}{c} s_{j}=\frac{1}{N_{v}\left({\mathrm{MB}}_{f}-1\right)}\;\sum_{u\in U_{j},u\neq j}\sum_{i=1}^{N_{v}}d_{i,u}\; \end{array}$$

where $N_{v}$ is the number of voxels in each slice. We also define the average global signal for all slices that were not acquired simultaneously to slice j as:

(7) $$\begin{array}{c} g_{U_{j}}=\frac{1}{N_{v}\left(N_{s}-{\mathrm{MB}}_{f}\right)}\sum_{j=1,j\notin U_{j}}^{N_{s}}\sum_{i=1}^{N_{v}}d_{i,j}\; \end{array}$$

To estimate the artifact signal via regression, we construct a design matrix:

(8) $$\begin{array}{c} X_{j}=\left[1\;g_{U_{j}}\;M\right] \end{array}$$

where 1 is a vector of 1s with length equal to the run timeseries (i.e., number of volumes), and M is a matrix of nuisance parameters, including six rigid‐body head motion parameters and their squares, derivatives, and squared derivatives. Next, we regress $X_{j}$ onto $s_{j}$:

(9) $$\begin{array}{c} b_{j}={\left({X_{j}}^{T}X_{j}\right)}^{-1}{X_{j}}^{T}s_{j}\; \end{array}$$

and estimate the artifact timeseries for slice j as the residuals of that regression, or:

(10) $$\begin{array}{c} a_{j}=s_{j}-X_{j}b_{j}\; \end{array}$$

#### 2.3.2 MARSS Removal of Artifact Signal

Given $a_{j}$, the artifact signal estimate for slice j, we construct a design matrix to estimate the artifact signal contribution to each voxel i in slice j:

(11) $$\begin{array}{c} W_{j}=\left[\begin{array}{ccc} 1 & \begin{array}{cc} a_{j} & g_{U_{j}} \end{array} & M \end{array}\right]\; \end{array}$$

and regress it onto the voxel‐wise timeseries $d_{i,j}$:

(12) $$\begin{array}{c} b_{i,j}={\left({W_{j}}^{T}W_{j}\right)}^{-1}{W_{j}}^{T}d_{i,j}\; \end{array}$$

The voxel‐wise artifact signal estimate $a_{i,j}$ can be defined as the product of the columns of $b_{i,j}$ and $W_{j}$ that correspond to the artifact estimate, $a_{j}$ (the second column):

(13) $$\begin{array}{c} a_{i,j}=b_{i,j_{a_{j}}}\times W_{j_{a_{j}}}\; \end{array}$$

Lastly, we obtain the corrected timeseries ${d^{\prime }}_{i,j}$ of voxel i in slice j as:

(14) $$\begin{array}{c} {d^{\prime }}_{i,j}=d_{i,j}-a_{i,j} \end{array}$$

Following MARSS correction, a new fMRI timeseries was saved as a NIFTI image, using the ${d^{\prime }}_{i,j}$ timeseries at each voxel in place of the original data. In addition, the spatial distribution of the artifact estimated by MARSS, A, was determined by taking the mean (over timepoints, t) of the absolute value of the artifact signal at each voxel:

(15) $$\begin{array}{c} A_{i,j}=\frac{1}{T}\sum_{t=1}^{T}\mathrm{abs}\left(a_{i,j,t}\right) \end{array}$$

#### 2.3.3 Alternative Artifact Estimation and Removal Methods

In a modification to the technique described above, artifact signal was estimated using only background signal. This was performed by masking out the brain prior to artifact estimation using whole‐brain masks generated by SynthStrip (Hoopes et al. 2022) included in FreeSurfer version 7.3.0 (Fischl 2012). Masks were dilated by one voxel prior to use. MARSS correction and removal remained otherwise unchanged in this modification.

Additionally, a two‐step MARSS procedure was developed in which artifact signal was estimated using only a percentage of voxels in each slice containing the highest MARSS artifact magnitude (as determined by a previous, unmodified MARSS correction procedure). In this procedure, the calculation of $s_{j}$ (see Equation 6) was modified to be the weighted average of voxel‐wise timeseries in slices acquired simultaneously to slice j that have artifact magnitudes above a given threshold $W_{j}$, weighted by the number of voxels above that threshold in each slice $w_{i}$:

(16) $$s_{j}^{*}=\frac{1}{\sum_{u\in U_{j},u\neq j}\sum_{i=1}^{N_{v}}w_{i}}\sum_{u\in U_{j},u\neq j\;}\sum_{i=1}^{N_{v}}w_{i,u}d_{i,u}$$

Thus, in the second step of this two‐step procedure, Equation (6) is replaced with Equation (16).

This procedure was tested in the NYSPI‐GE task‐based dataset using the top 30% (MARSS30), 20% (MARSS20), and 10% (MARSS10) of voxels with the highest artifact magnitude. The 30% threshold was chosen such that each slice across the entire dataset would have at least 75 voxels contributing to the mean timeseries in that slice. The 20% and 10% thresholds were chosen to further evaluate how many voxels were required across a run to adequately model and remove the artifact signal.

### 2.4 Characterization of Isolated Artifact Signal

Following MARSS artifact estimation and removal, we sought to investigate the spectral, temporal, and spatial properties of the isolated artifact signal to provide evidence for its potential source. Several of these analyses are described in the Data S1, including calculation of power spectral density (PSD), autocorrelation as a function of lag, the association between artifact magnitude and measures of in‐scanner motion, the effects of slice timing correction (STC) on artifact magnitude, and characterization of artifact magnitude in gray matter, white matter, and cerebrospinal fluid (CSF).

#### 2.4.1 Interslice Leakage Artifact Analysis

Interslice leakage artifacts have been previously shown to contaminate estimates of neural signal in MB fMRI (McNabb et al. 2020). They arise as a consequence of the imperfect “unmixing” of signals in simultaneously acquired slices that are originally stacked and phase‐shifted by a set fraction of the field of view (FOV) immediately preceding reconstruction. Interslice leakage artifacts, however, typically comprise a shared signal between two voxels that were overlaid during acquisition, rather than entire slices. We sought to demonstrate that the artifact detected and removed by MARSS is not a product of interslice leakage.

In resting‐state MB fMRI data in the SBU‐Siemens dataset, it was known that simultaneously acquired slices were phase‐shifted by multiples of $\frac{\mathrm{FOV}}{3}$ voxels (i.e., multiples of 34 voxels) relative to each other prior to reconstruction. For each voxel, the contribution to its signal due to the voxels corresponding to the phase shift was estimated and removed via regression; that is, each voxel in the image was subjected to a regression in which the signals in all voxels that were “stacked” on top of that voxel were regressed out. Although this is destructive to the true underlying neural signal in each voxel, it was employed as a method to determine whether the MARSS artifact remained following removal of slice leakage effects from the data. The presence of the shared artifact in simultaneously acquired slices that remained after removing any potential interslice leakage was then determined using prior methods (see above, Section 2.2). It is important to note that this procedure is not intended to be a step of MARSS, but rather a separate procedure to demonstrate that the MARSS‐corrected artifact signal is independent of interslice leakage.

Additionally, we calculated the Pearson correlation between timeseries in voxels of simultaneous slices that were expected to overlap due to the phase shift during image reconstruction. This was done prior to and following MARSS correction, as well as in data that had undergone slice leakage regression but not MARSS correction, to further demonstrate that MARSS is removing artifact signal independent of interslice leakage.

#### 2.4.2 Analysis of Artifact Spatial Distribution

We assessed the average spatial distribution of the isolated artifact signal in resting‐state data in NYSPI‐GE, SBU‐Siemens, and HCP‐Siemens. For each run, the MARSS estimate of the 3D spatial distribution of artifact was obtained using Equation (15), above. Each run's spatial distribution was warped and resampled into MNI152Nlin6Asym template space (Grabner et al. 2006) by the single‐step resampling procedure within the HCP Minimal Preprocessing Pipeline (OneStepResampling.sh; Glasser et al. 2013). This was performed after fMRI preprocessing and utilized previously calculated gradient distortion corrections and affine transformations calculated during that process (see below, Section 2.6.3). To determine how the magnitude of signal fluctuations due to artifact was impacted by the underlying signal intensity (e.g., brain matter versus empty space), this process was repeated after rescaling each unprocessed average artifact distribution to reflect the percentage of artifact signal relative to mean signal intensity on a per‐voxel basis.

#### 2.4.3 Temporal Signal‐To‐Noise Ratio

Following artifact removal, we assessed the resulting change in whole‐brain temporal signal to noise ratio (tSNR) in both unprocessed and preprocessed resting‐state and task‐based data. For each run, tSNR in voxel i of slice j was calculated as:

(17) $$\begin{array}{c} {\text{tSNR}}_{i,j}=\frac{\;\bar{s_{i,j}}}{\sigma_{i,j}} \end{array}$$

where $\bar{s_{\mathrm{ij}}}$ is the mean signal and $\sigma_{\mathrm{ij}}$ is the standard deviation of the signal (Murphy, Bodurka, and Bandettini 2007; Welvaert and Rosseel 2013). In unprocessed data, voxel‐wise tSNR estimates were averaged within dilated, whole‐brain masks generated for each run by SynthStrip (Hoopes et al. 2022) and compared between corrected and uncorrected data. In preprocessed data, mean tSNR difference and percent difference maps were generated separately for resting‐state and task‐based data in NYSPI‐GE and SBU‐Siemens. Significance of the elevation in tSNR across each dataset was determined via Wilcoxon signed‐rank tests.

#### 2.4.4 Comparison to Physiological Correction via RETROICOR

In order to evaluate whether the artifact signal isolated by MARSS could be explained by physiological motion, we performed RETROICOR (Glover, Li, and Ress 2000) in the HCP‐Siemens resting‐state data using each run's publicly available cardiac and respiratory traces. For each run, 18 RETROICOR regressors, comprising a third‐order cardiac model (6 regressors), fourth‐order respiration model (eight regressors), and first‐order cardiac*respiration interaction terms (four regressors), were estimated using the PhysIO Toolbox (Kasper et al. 2017) integrated with SPM12 (Friston et al. 2004, 2007). Signal contributions related to each regressor were subsequently removed from each run via regression. This procedure was repeated in data that had previously been MARSS corrected, and artifact presence was evaluated in each (see above, Section 2.2).

Additionally, the spatial distributions of signal removed via RETROICOR alone and RETROICOR following MARSS correction were obtained for each run using Equation (15) above. Signal in each slice was averaged across the x‐ and y‐ dimensions, resulting in a single value per slice, and mean‐centered. Frequency spectral analysis was then performed using methods described in the Data S1.

Spatial distributions of signal removed via RETROICOR alone were also warped and resampled (see above, Section 2.4.2) in order to be directly compared to the MARSS artifact spatial distribution.

### 2.5 Evaluation of Denoising via sICA+FIX

Since we do not intend for MARSS to be a standalone denoising solution for multiband fMRI, we investigated its efficacy alongside sICA+FIX denoising (Griffanti et al. 2014; Salimi‐Khorshidi et al. 2014), which is currently implemented in all publicly available, HCP resting‐state data (Glasser et al. 2013; Hodge et al. 2016; Ugurbil et al. 2013; Van Essen et al. 2013). We were particularly interested in the magnitude of the observed artifact in simultaneously acquired slices remaining in the data following sICA+FIX denoising.

#### 2.5.1 Implementation of sICA+FIX in Unprocessed, Resting‐State Data

For each resting‐state run in the HCP‐Siemens dataset, unprocessed fMRI data and associated motion parameters, derivatives, squares, and squared derivatives were first linearly detrended. Independent component (IC) regressors were taken from the previously calculated mixing matrix (melodic_mix) released with the Resting State fMRI 1 FIX‐Denoised (Extended) and Resting State fMRI 2 FIX‐Denoised (Extended) packages from ConnectomeDB (Hodge et al. 2016). IC regressors were then z‐scored, and the 24 detrended motion parameters were regressed out of both the mixing matrix and the detrended fMRI data. FIX‐cleaned data was obtained by regressing all IC regressors onto the fMRI data and calculating the residuals with respect to noise components, only. This procedure was performed in both uncorrected, unprocessed data and MARSS‐corrected data.

#### 2.5.2 Subspace Decomposition of sICA+FIX Denoised Data

Denoising via sICA+FIX estimates and removes structured noise components but does not model unstructured or random noise components. These unmodeled components will contain mostly Gaussian noise, but may also contain some weakly structured signals that were not successfully modeled by sICA (Glasser et al. 2018). Therefore, the denoised data can be decomposed to a sum of a “neural signal” subspace and an unstructured, “random noise” subspace. We performed this decomposition following methods described elsewhere (see Supporting Information in Glasser et al. 2018) to determine which data subspace contains artifactually elevated correlations between simultaneously acquired slices that are corrected by MARSS. The random noise subspace was obtained by residualizing uncorrected data with respect to all IC regressors, regardless of their classification. The neural signal subspace was then obtained by subtracting the random noise subspace from the sICA+FIX denoised data (i.e., data in which IC regressors classified as noise components are removed). In addition to this decomposition, the signal estimated by sICA+FIX noise components was also evaluated for simultaneous slice artifact presence. Finally, global signal regression (GSR) was performed on the neural subspace to determine whether the residual artifact in this subspace could be captured as global structured noise or signal (Glasser et al. 2018). Global signal was estimated as the mean timeseries across all in‐brain voxels, as determined by previously generated brain masks (see above, Section 2.3.3).

In each subspace, artifact magnitude was assessed using methods described previously (see above, Section 2.2), with the only modification being that correlations were only calculated between slices in which all runs of all participants contained in‐brain voxels. Correlation matrices of the difference between uncorrected and MARSS‐corrected subspaces were also calculated to determine whether structured correlations beyond those in simultaneously acquired slices, specifically those that could reflect neural signal, were being removed by MARSS.

Following the calculation of data subspaces, we performed a variance decomposition of the raw data prior to and following MARSS correction, which is described in detail in the Data S1.

### 2.6 Task‐Based Analysis

We sought to determine the effects of MB artifact correction on the spatial distribution of statistical maps resulting from task‐based modeling (see Data S1). We first performed three within‐participants analyses on unprocessed, working memory task‐based MB fMRI data in NYSPI‐GE (*n* = 58), SBU‐Siemens (*n* = 10), and HCP‐Siemens (*n* = 25) before and after MARSS correction. We then performed separate between‐participants analyses on preprocessed SBU‐Siemens and NYSPI‐GE data to highlight the effects of artifact correction on cortical, group‐level *t*‐statistics.

#### 2.6.1 Within‐Participants Task Modeling

Prior to modeling, each fMRI run was normalized to reflect percent signal change on a per‐voxel basis, $n_{i,j}=100\cdot s_{i,j}/\bar{s_{i,j}}$, where $s_{i,j}$ is the timeseries and $\bar{s_{i,j}}$ is the mean signal in voxel i in slice j. Each task was modeled using a single‐parameter canonical hemodynamic response function (HRF) in SPM12 (Friston et al. 2004, 2007). For the SOT, regressors of interest were those reflecting correct trials in the control task or each of the first seven steps of the SOT, modeled separately. For the visual n‐back task, a block design was utilized to model all 0‐back and 2‐back conditions. Trial timings were extracted from event files (“EVs”) distributed with HCP task data. Nuisance parameters included six motion parameters, their squares, derivatives, and squared derivatives, as well as spike regressors to remove high motion volumes, without being convolved with the HRF. Spike regressors were identified using run‐adaptive, generalized extreme value, low‐pass filtered DVARS (GEV‐DV) thresholds and chosen to identify approximately 3% of total volumes across each dataset (Williams et al. 2022). The GEV‐DV parameters used for each dataset were 28 in NYSPI‐GE, 21 in SBU‐Siemens, and 42 in HCP‐Siemens.

Following model estimation, whole‐brain mean squared residuals were obtained for each participant as a measure of within‐participant model error and compared in uncorrected and MARSS‐corrected data via Wilcoxon signed‐rank test. Additionally, the Bayesian Information Criterion (BIC; Bollen et al. 2014; Hastie, Tibshirani, and Friedman 2009; Schwarz 1978) was calculated in uncorrected and MARSS‐corrected data as a measure of overall model fit that accounts for the loss of one statistical degree of freedom due to MARSS correction. BIC was calculated as follows:

(18) $$\mathrm{BIC}=N\times \ln\left(2\pi \right)+N+N\times \ln\left(\frac{\text{resMS}}{N}\right)-\mathrm{DOF}\times \ln\left(N\right)$$

where N is the overall number of volumes across runs for each subject, resMS is the mean‐squared residuals obtained from within‐participants analysis, and DOF is the number of degrees of freedom; MARSS‐correction reduces DOF by one.

#### 2.6.2 Spatial Frequency Analysis of Unprocessed Task fMRI

We performed a spatial frequency analysis along the slice direction in each task dataset to evaluate the presence of periodic patterns of task betas corresponding to simultaneous slice spacing both before and after artifact removal. This was performed separately for task and control conditions in each task. One participant in SBU‐Siemens and seven participants in NYSPI‐GE were excluded from this analysis (and subsequent analyses of preprocessed data; see below, Section 2.6.4) for not sufficiently performing all task conditions. Betas for each condition were averaged across the x‐ and y‐ dimensions, resulting in a single value per slice, and mean‐centered. Frequency spectral analysis was then performed using previously described methods (see above, Data S1). In the NYSPI‐GE dataset, this analysis was also performed in data corrected via MARSS30, MARSS20, and MARSS10.

#### 2.6.3 fMRI Preprocessing

Corrected and uncorrected data in SBU‐Siemens and NYSPI‐GE were preprocessed using the HCP Minimal Preprocessing Pipeline (HCP MPP; Glasser et al. 2013; Ugurbil et al. 2013) Version 4.2.0. Briefly, data underwent processing through the PreFreeSurfer, FreeSurfer, PostFreeSurfer, fMRIVolume, and fMRISurface pipelines. A critical step in the HCP preprocessing is its ribbon‐constrained volume to surface mapping algorithm (Glasser et al. 2013). Notably, this step chooses to exclude voxels with locally high coefficients of variation (COV) from the mapping process. We quantified the mean, voxel‐wise change and percent change in COV before and after MARSS correction within the cortical ribbon isolated for each participant during anatomical volume preprocessing to determine whether MARSS increases the probability that cortical voxels will be included in volume to surface mapping.

Using the preprocessed outputs in CIFTI format (dtseries.nii), cortical surface GIFTI files and subcortical NIFTI files were generated, smoothed with a 4 mm full width at half maximum Gaussian kernel, and normalized to reflect percent signal change on a per‐greyordinate basis.

#### 2.6.4 Between‐Participants Analysis

First level modeling was performed for the SOT as previously described (see above, Section 2.6.1), with the exception of using cortical surfaces and subcortical volumes rather than pure volumetric data. Task contrasts for each participant were calculated as the average beta estimate from the first seven steps of the SOT minus the control condition.

Contrasts from corrected and uncorrected data were analyzed separately in between‐participants analyses using Permutation Analysis of Linear Models (PALM; Winkler et al. 2016; Winkler et al. 2014; Winkler et al. 2016; Winkler et al. 2015) alpha119. Analyses in PALM were performed using 1024 and 20,000 sign flips in SBU‐Siemens and NYSPI‐GE, respectively, both with and without threshold‐free cluster enhancement (TFCE; Smith and Nichols 2009). *t*‐statistic result images with and without TFCE were subtracted to obtain differences in *t*‐statistics resulting from artifact removal. This subtraction was also repeated after taking the absolute value of *t*‐statistics in corrected and uncorrected data where uncorrected data had negative *t*‐statistics, yielding difference maps reflecting the magnitude of the change in *t*‐statistics (i.e., so that a decrease in signal in an area showing deactivation would appear as an increase in *t*‐statistic magnitude), rather just the direction of the change.

In addition to this, we quantified the change in significant greyordinates resulting from MARSS correction. *t*‐statistic maps were thresholded at *p* < 0.05, Šidák‐corrected (Šidák 1967), and the number of significant greyordinates prior to and following MARSS correction were calculated, as well as the number of greyordinates that crossed the threshold for significance as a result of MARSS correction. Spatial similarity of thresholded *t*‐statistic maps was characterized using Sørensen–Dice coefficients (Dice 1945; Sørensen 1948).

Finally, we quantified the change in *t*‐statistic within an a priori region of interest (ROI), the left dorsolateral prefrontal cortex (lDLPFC). The lDLPFC ROI (Figure S1) was derived from regions of significant activation in response to SOT task demands from previous work (Van Snellenberg et al. 2016). The ROI was first resampled from volume to surface space, then dilated to fill holes, and finally eroded by 4 mm. This analysis was performed on between‐participant cortical *t*‐statistic maps generated both with and without the use of TFCE.

## 3 Results

### 3.1 Artifact Identification and Removal Across Multiple Datasets

Elevated Pearson correlations in simultaneously acquired slices over adjacent‐to‐simultaneous slices across multiple task‐based, in vivo datasets can be seen in correlation matrices in Figure 1; (Table S3). Diagonal, striped patterns of elevated Pearson correlations were observed in all other in vivo and phantom datasets explored regardless of scanner platform or task type (Figures S2 and S3; Table S4). Notably, the pattern of elevated correlations is directly related to the MB factor used during acquisition (see Figure S2). Following MARSS correction, correlations in simultaneously acquired slices were significantly reduced to values closer to the mean correlation between adjacent‐to‐simultaneous slices (Table S5 for statistics). As a baseline comparison to various slice correlation structures seen across MB fMRI datasets, slice correlations matrices from the NYSPI‐Philips and QTIM‐Bruker single‐band datasets, both prior to and following STC, can be seen in Figure S4.

Results of alternative artifact removal procedures tested in SBU‐Siemens resting‐state data are shown in Figures S5 and S6. When artifact magnitude was estimated using background alone, the difference in correlation between simultaneously acquired slices and adjacent‐to‐simultaneous slices was reduced from 0.399 to 0.309 (*W*(42) = 903, *p* = 1.648 × 10^−8^; see Figure S5). This decrease in correlation, however, was much less pronounced than that produced by standard MARSS artifact correction.

Regression‐based interslice leakage removal produced a slight increase in the elevation of correlations between simultaneously acquired over adjacent‐to‐simultaneous slices, from 0.399 to 0.405 (*W*(42) = 213, *p* = 0.029; see Figure S6). This indicates that no level of artifact correction was achieved by this method. Figure S7 shows correlations between voxel pairs in simultaneously acquired slices as a function of the phase shift between the voxels during multiband reconstruction in SBU‐Siemens resting‐state data. Regression‐based interslice leakage removal reduces the mean correlation at the expected phase shift of $\frac{\mathrm{FOV}}{3}$ voxels (or 34 voxels) from 0.0559 in uncorrected data to −0.0273. In contrast, MARSS correction produces only a slight decrease in correlation between these voxels from 0.0559 to 0.0548.

**FIGURE 2:**
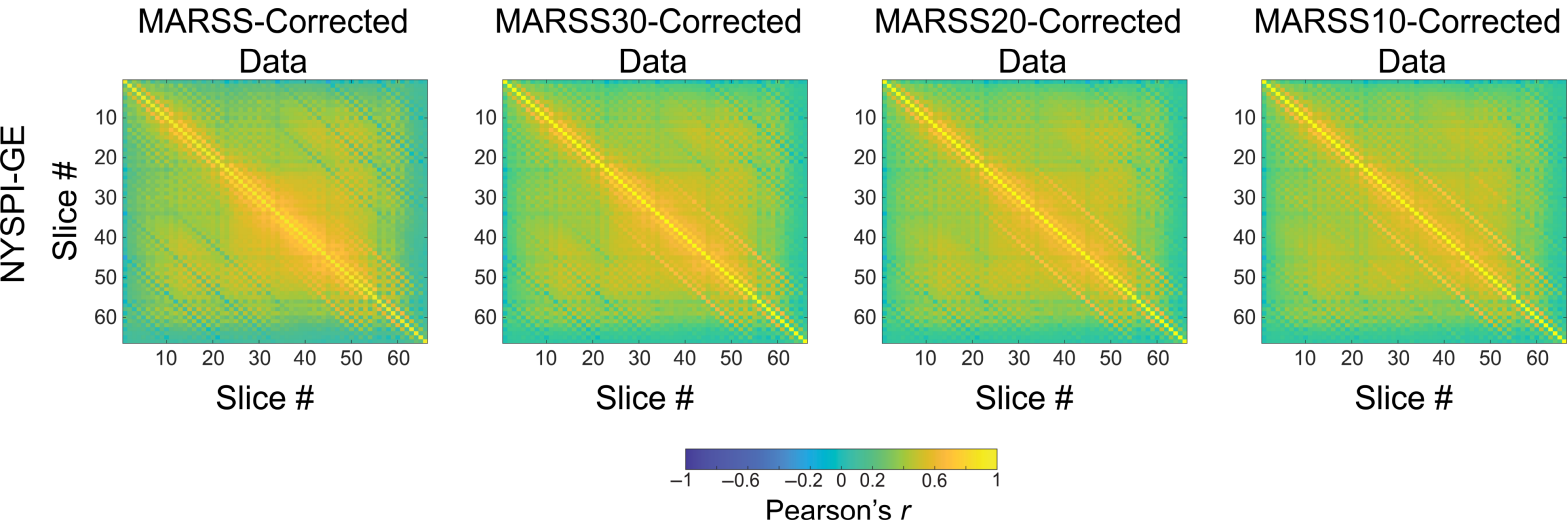
Pearson correlation matrices between average signal in all slice pairs in MARSS‐corrected data, as well as data corrected with MARSS procedures with artifact signal intensity thresholds (MARSS30, MARSS20, and MARSS10), from the NYSPI‐GE task‐based dataset (*N* = 58).

Slice correlation matrices resulting from MARSS30, MARSS20, and MARSS10 in the NYSPI‐GE task‐based dataset can be seen in Figure 2. Compared to standard MARSS correction, MARSS30 reduced the magnitude of the negative difference in Pearson correlation between simultaneously acquired slices and adjacent‐to‐simultaneous slices from −0.085 to −0.024 (Table S6). MARSS20 resulted in a slight positive correlation difference between simultaneously acquired slices and adjacent‐to‐simultaneous slices of 0.016. MARSS10 resulted in a positive correlation difference between simultaneously acquired slices and adjacent‐to‐simultaneous slices of 0.086, which is nearly equivalent in magnitude to the negative correlation difference induced by standard MARSS correction.

Finally, the application of STC to the SBU‐Siemens resting‐state data did not produce a significant change in the difference in slice correlations between simultaneously acquired and adjacent‐to‐simultaneous slices (*W*(42) = 388, *p* = 0.427; see Figure S8).

### 3.2 Characterization of Artifact Signal

#### 3.2.1 Spatial Artifact Distribution

**FIGURE 3:**
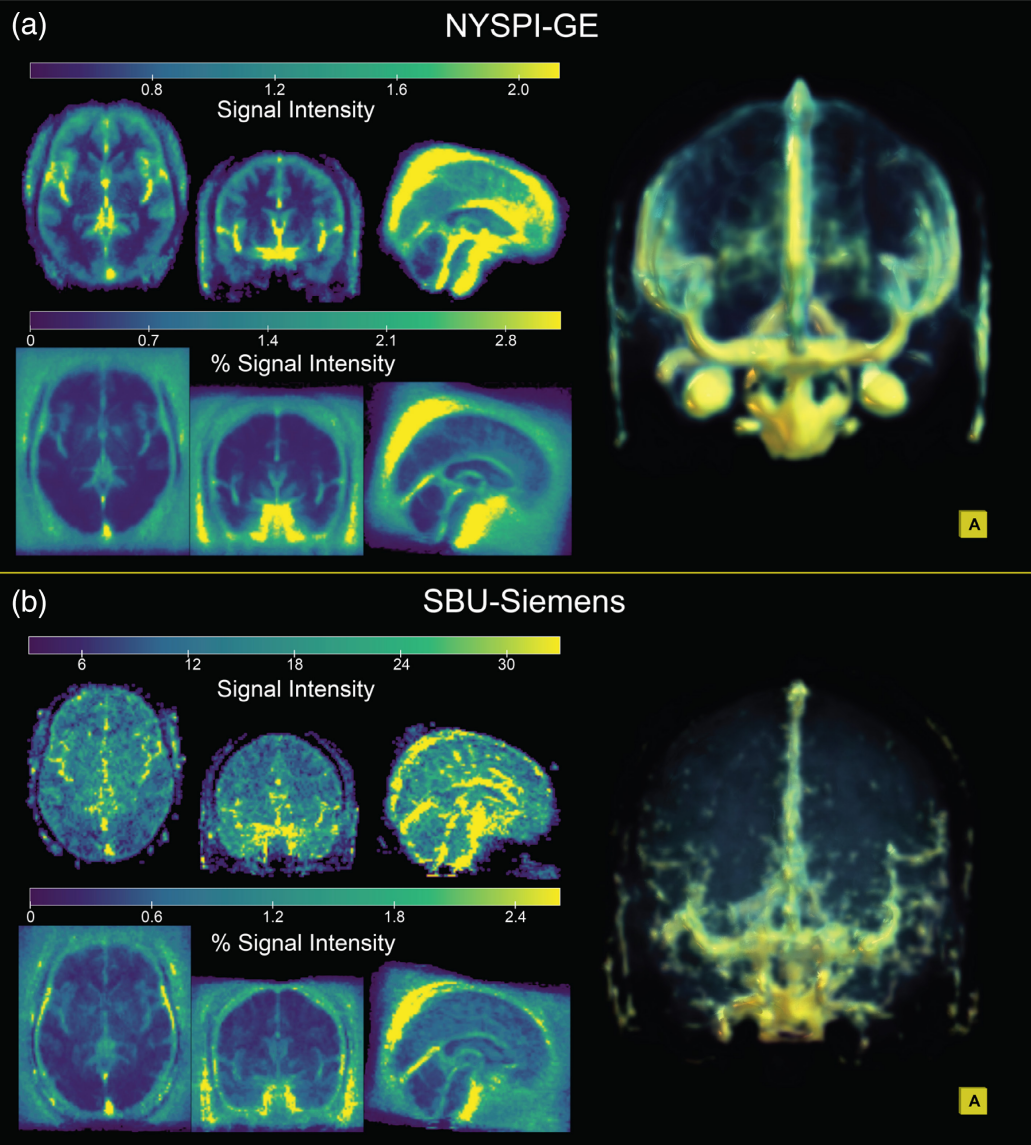
Average spatial distribution of the isolated artifact signal in resting‐state data from (a) NYSPI‐GE (*N* = 63) and (b) SBU‐Siemens (*N* = 10). Artifact distribution is shown as both raw EPI signal intensity (top left of each panel) and as a percentage of the mean voxelwise EPI intensity (bottom left of each panel). Three‐dimensional renders (right of each panel) display anterior view of the raw signal intensity artifact distribution.

The spatial distributions of the average MARSS‐estimated artifact signal across resting‐state runs in NYSPI‐GE and SBU‐Siemens are shown in Figure 3. Images are shown on both the scale of the raw EPI data and as a percentage of the mean voxel‐wise EPI signal intensity. A video depicting a 360° view of the rendered NYSPI‐GE spatial distribution is included in the Video S1. Artifact signal appears strongest in vasculature, notably the superior sagittal sinus, inferior sagittal sinuses, transverse sinuses, middle cerebral arteries, Circle of Willis, and superficial temporal arteries, as well as the eyes. Further, MARSS removes a larger percentage of voxel signal from both the image background and vasculature than from gray and white matter.

The spatial distribution of the MARSS‐estimated artifact signal in a single phantom run from SBU‐Siemens acquired with a MB factor of 6 is shown in Figure S9. Artifact signal is strongest near the posterior edge of the phantom, where it contacted the scanner table.

#### 3.2.2 Spectral and Autocorrelative Properties

PSD estimates and autocorrelations of the MARSS‐estimated artifact signal timeseries across all in vivo, task‐based fMRI datasets are shown in Figure S10. All PSD estimates were linearly fit with slopes between −0.716 and − 0.935 (Figure S10, top row). Autocorrelations in each dataset show varying degrees of long‐range dependence from approximately 5 to 15 lags (Figure S10, bottom row). These spectral and autocorrelative characteristics roughly correspond to the properties expected of 1/f colored noise.

**FIGURE 4:**
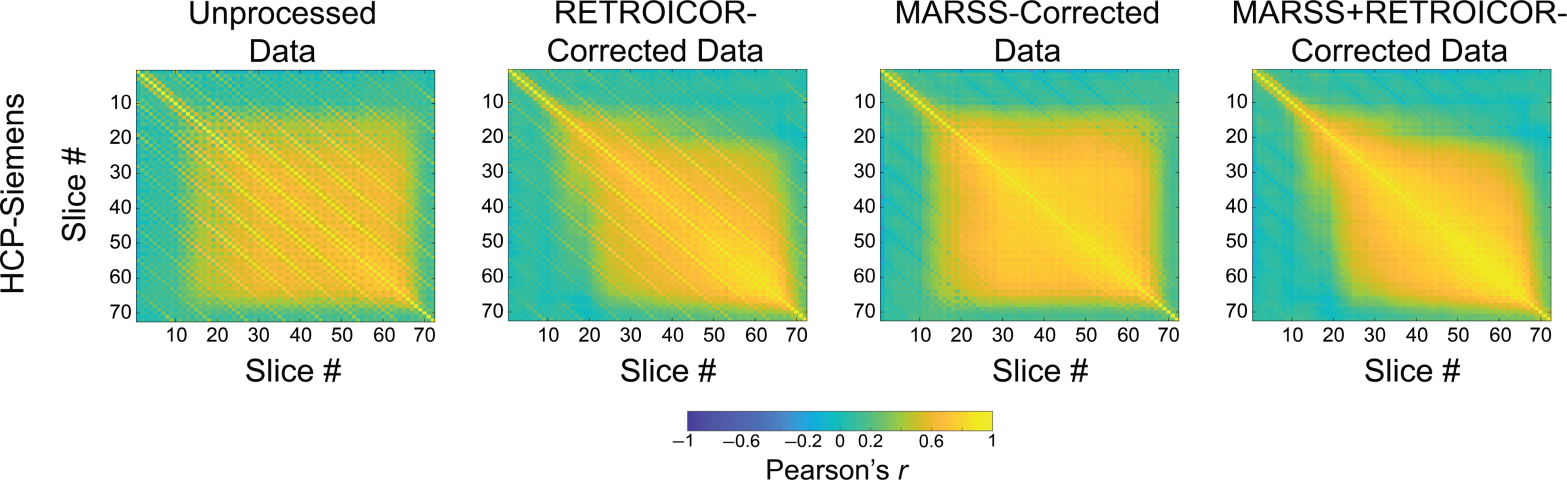
Pearson correlation matrices between average signal in all slice pairs in the HCP‐Siemens resting‐state dataset (*N* = 25) in fully uncorrected data, data that has undergone regression‐based removal of RETROICOR predictors, MARSS‐corrected data, and data that has undergone MARSS correction prior to RETROICOR.

Additional PSD estimates are shown from all in vivo, resting‐state fMRI datasets in Figure S11, and phantom data acquired with an MB factor of 6 at SBU in Figure S12. Autocorrelation of the artifact timeseries in in vivo, resting‐state data as a function of lag is shown in Figure S13.

#### 3.2.3 Associations Between Artifact Magnitude and in‐Scanner Motion

Associations between mean and median measures of in‐scanner motion and artifact magnitude, measured as the elevation of signal correlation between simultaneously acquired slices over adjacent‐to‐simultaneous slices, in NYSPI‐GE resting‐state and task‐based data are shown in Figure S14. All motion measures except median DV in the NYSPI‐GE resting‐state dataset were significantly positively associated with artifact magnitude (all *p* < 0.05).

#### 3.2.4 Temporal Signal‐To‐Noise Ratio

Across all unprocessed, in vivo datasets, 100% of runs showed significant increases in mean, whole‐brain tSNR (all *p* < 0.001; Table S7) following MARSS correction. In preprocessed, task‐based data, 69.91% of voxels in NYSPI‐GE and 95.16% of voxels in SBU‐Siemens experienced mean tSNR increases across participants (Figure S15, top row; see Figure S16 for percent change in tSNR). In preprocessed, resting‐state fMRI data, 77.79% of voxels in NYSPI‐GE and 97.45% of voxels in SBU‐Siemens experienced mean tSNR increases (Figure S17; see Figure S18 for percent change in tSNR).

#### 3.2.5 MARSS Correction in Gray Matter, White Matter, and Cerebrospinal Fluid

Slice correlation matrices calculated from voxels containing gray matter, white matter, and CSF in the NYSPI‐GE task‐based and resting‐state datasets can be seen in Figures S19 and S20, respectively. MARSS correction leads to decreases in elevated correlations between simultaneously acquired slices in all tissue compartments, with the most pronounced decrease observed in gray matter (see Tables S8 and S9). Notably, no negative correlation differences between simultaneously acquired slices and adjacent‐to‐simultaneous slices were induced. Further, increases in mean tSNR were observed in all tissue compartments (see Table S10). The largest mean artifact magnitude existed within CSF, with gray matter containing the second largest.

#### 3.2.6 Comparison to Physiological Correction via RETROICOR

Slice correlation matrices from unprocessed, HCP‐Siemens resting‐state data that has undergone physiological correction via RETROICOR alone, MARSS correction alone, and MARSS correction followed by RETROICOR can be seen in Figure 4. RETROICOR produces a reduction in the difference in correlation between simultaneously acquired slices and adjacent‐to‐simultaneous slices from 0.414 to 0.222 (see Table S11; *W*(25) = 4954, *p* = 6.729 × 10^−17^), but the magnitude of reduction does not match that induced by MARSS alone. The average spatial distribution across participants of signal removed via RETROICOR alone versus MARSS alone is shown in Figure S21. MARSS removes a globally higher signal magnitude than RETROICOR, and clearly targets major neurovasculature to a greater extent than RETROICOR.

Finally, the PSD estimates of the average spatial distribution of signal removed by RETROICOR alone and RETROICOR following MARSS correction as a function of spatial frequency are shown in Figure S22. Without MARSS, signal isolated by RETROICOR shows elevated PSD at the fundamental and harmonic frequencies associated with the slice acquisition pattern. However, RETROICOR following MARSS correction does not isolate any of these frequencies except for the final harmonic frequency of 0.278 mm^−1^, suggesting that MARSS correction previously removed artifactual signal at those frequencies.

### 3.3 Evaluation of Artifact Magnitude in sICA+FIX Denoised Data Subspaces

**FIGURE 5:**
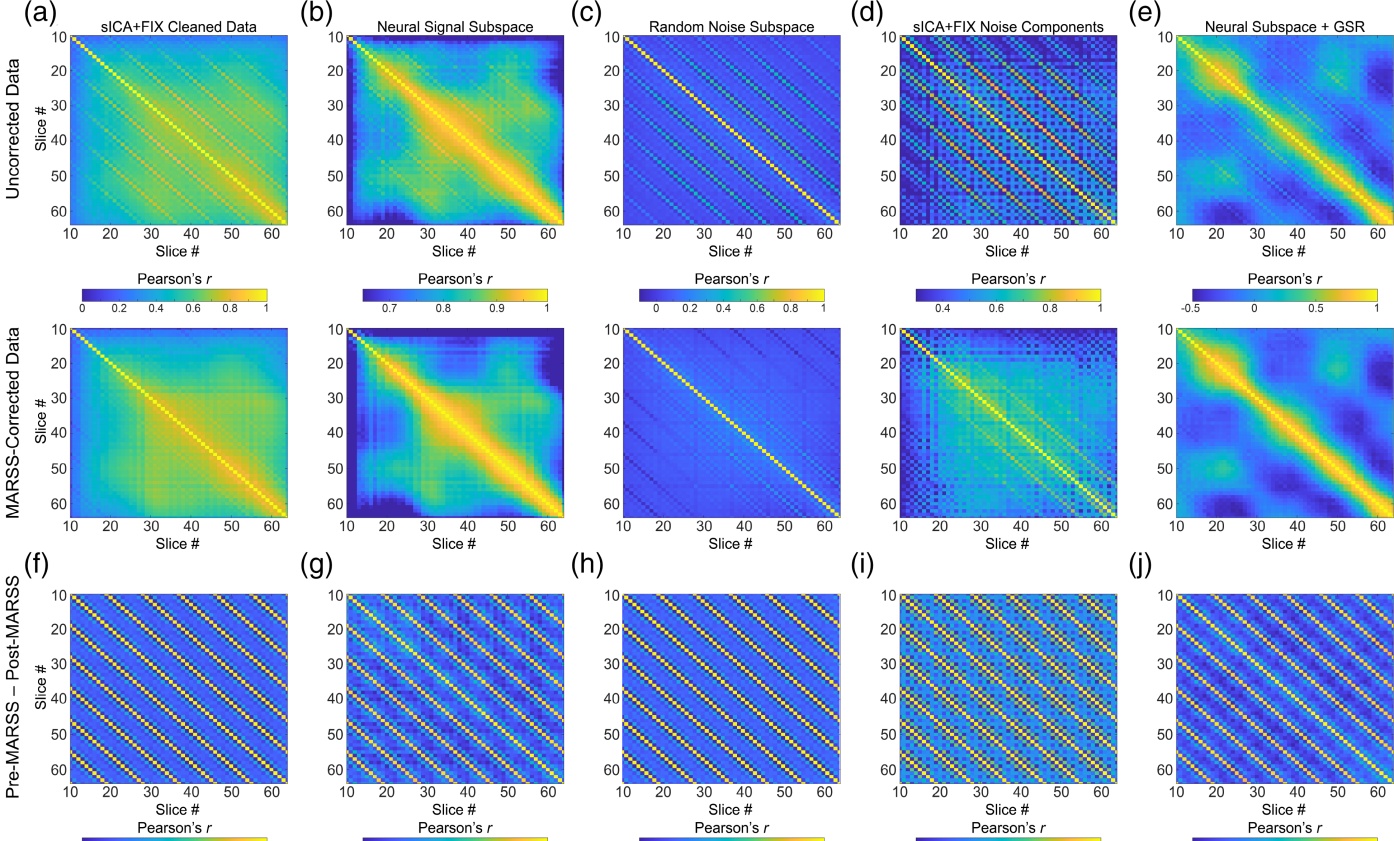
Pearson correlation matrices between average signal of all in‐brain slice pairs of (a) sICA+FIX cleaned data, (b) the Neural Signal Subspace of sICA+FIX cleaned data, (c) the Random Noise (or unstructured noise) of sICA+FIX cleaned data, (d) Noise Components removed via sICA+FIX, and (e) the Neural Signal Subspace after global signal regression (GSR). Correlations are only shown between slices that contain in‐brain voxels across all participants. Analyses were performed in resting‐state data from the HCP‐Siemens dataset (*N* = 25) both prior to and following MARSS correction. Panels (f–j) show Pearson correlation matrices of the difference between uncorrected and MARSS‐corrected data, corresponding to panels (a–e), respectively.

The average slice correlation matrices in sICA+FIX denoised data, the neural subspace, random noise subspace, sICA+FIX noise components, and neural subspace with GSR, as well as correlation matrices for the difference between uncorrected and MARSS‐corrected data, are shown in Figure 5. In uncorrected data, elevated correlations between simultaneously acquired slices over adjacent‐to‐simultaneous slices are observable in all data subspaces (all *p* < 0.05, see Table S12); the correlation differences between simultaneously acquired slices and adjacent‐to‐simultaneous slices in sICA+FIX cleaned data and the neural subspace are 0.176 and 0.081, respectively. MARSS correction led to significant reductions in correlation differences in all subspaces (all *p* < 0.05). Correlation matrices of the difference between uncorrected and MARSS‐corrected data contain correlation elevations of simultaneously acquired slices over adjacent‐to‐simultaneous slices above 0.9 (see Table S12).

Following MARSS correction, a statistically significant difference in correlations between simultaneously acquired slices and adjacent‐to‐simultaneous slices of −0.004 was observed in the neural subspace following GSR (*W*(100) = 1795, *p* = 0.0121). Additionally, a correlation difference of 0.022 was observed in the sICA+FIX noise components (*W*(100) = 4150, *p* = 2.307 × 10^−8^). No significant correlation differences were observed in the sICA+FIX cleaned data (*W*(100) = 2165, *p* = 0.2158), neural signal subspace (*W*(100) = 1970, *p* = 0.0564), and random noise subspace (*W*(100) = 2382, *p* = 0.6229).

Results of a full variance decomposition of uncorrected and MARSS‐corrected data following sICA+FIX denoising can be seen in Table S13 and are discussed in Data S1.

### 3.4 Within‐Participants Analysis of Unprocessed Task fMRI

**FIGURE 6:**
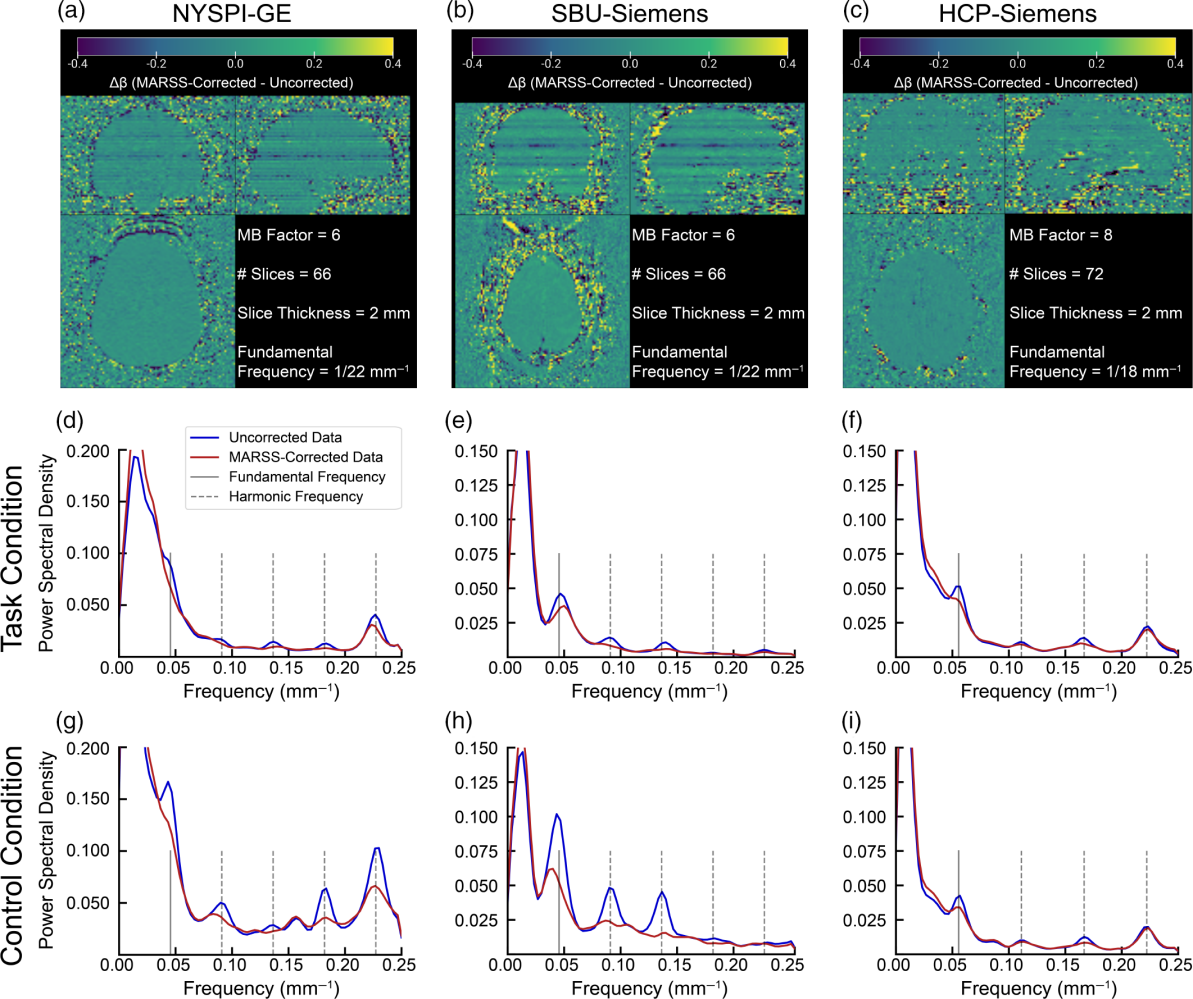
Single‐participant examples of the spatial distribution of differences in task betas between MARSS‐corrected and uncorrected data (a–c), as well as power spectral density (PSD) of average within‐participants betas as a function of spatial frequency (d–i) in uncorrected data (blue line) and corrected data (red line) in data from NYSPI‐GE (*N* = 52; A, D, G), SBU‐Siemens (*N* = 9; B, E, H), and HCP‐Siemens (*N* = 25; F, I). In PSD plots, vertical lines denote the fundamental spatial frequency (solid gray line) and harmonic spatial frequencies (dotted gray lines) associated with the simultaneous slice pattern in each dataset. PSD is calculated separately for the task‐on condition (d–f) and control condition (g–i) of each task.

Single‐participant examples of the difference in within‐participant betas in unprocessed data from the task‐on condition before and after MARSS correction are shown in Figure 6 (top row). In all datasets, the spatial pattern of beta differences is systematic and occurs at both the fundamental spatial frequency of simultaneously acquired slice spacing and its corresponding harmonic spatial frequencies. There is a reduction in PSD at these spatial frequencies in all datasets following MARSS (Figure 6, bottom row). Additionally, MARSS30, MARSS20, and MARSS10 produce comparable reductions in PSD at the fundamental and harmonic spatial frequencies in the NYSPI‐GE dataset (see Figure S23).

### 3.5 Within‐ and Between‐Participants Analysis of Preprocessed Task fMRI

#### 3.5.1 MARSS Correction Effects on Volume to Surface Mapping

Decreases in cortical COV were seen in 94.62% of task‐based runs in NYSPI‐GE (*W*(186) = 534, *p* = 1.247 × 10^−28^) and 100% of task‐based runs in SBU‐Siemens (*W*(39) = 0, *p* = 5.255 × 10^−8^) following MARSS, with 80.77% of voxels in NYSPI‐GE and 97.0% of voxels in SBU‐Siemens experiencing mean decreases across participants (Figure S15, bottom row; see Figure S16 for percent change in COV).

#### 3.5.2 MARSS Correction Effects on Within‐Participants Modeling

Following MARSS, we observe a mean decrease in within‐participants mean squared residuals of −0.045 (−2.16%) in NYSPI‐GE (*W*(52) = 0, *p* = 3.504 × 10^−10^) and − 0.062 (−5.3%) in SBU‐Siemens (*W*(10) = 0, *p* = 0.002). Additionally, we observe a mean decrease in BIC of −48.412 in NYSPI‐GE (*W*(52) = 0, *p* = 3.504 × 10^−10^) and − 173.799 in SBU‐Siemens (*W*(10) = 0, *p* = 0.002), indicating better whole‐brain model fit following MARSS.

#### 3.5.3 Between‐Participants Analysis

**FIGURE 7:**
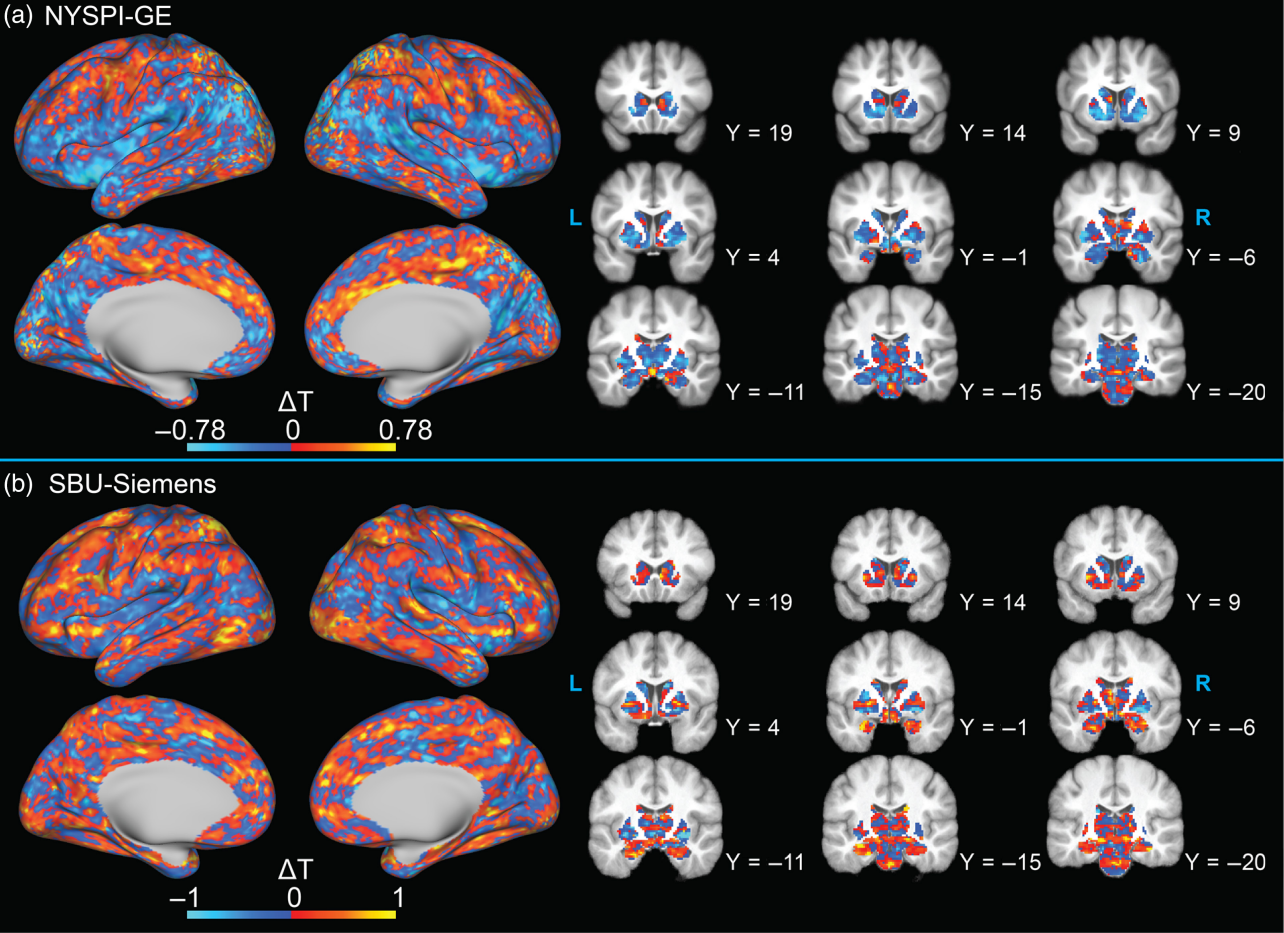
Spatial distribution of the difference in *t*‐statistics in between‐participants analyses performed in MARSS‐corrected and uncorrected data from (a) NYSPI‐GE (*N* = 52) and (b) SBU‐Siemens (*N* = 9). *t*‐statistics were obtained without the use of threshold‐free cluster enhancement (TFCE).

**FIGURE 8:**
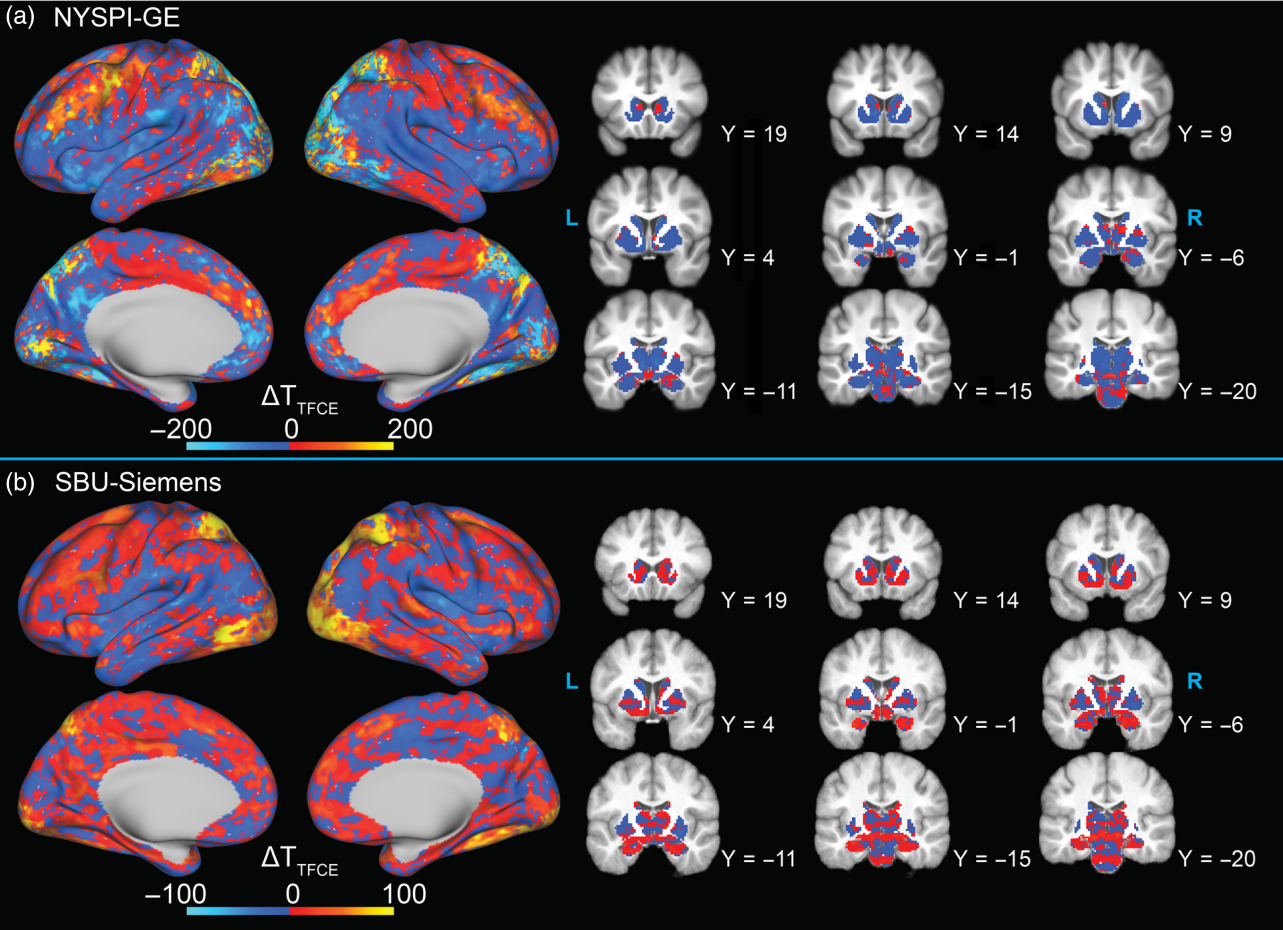
Spatial distribution of the difference in *t*‐statistics in between‐participants analysis performed in MARSS‐corrected and uncorrected data from (a) NYSPI‐GE (*N* = 52) and (b) SBU‐Siemens (*N* = 9). *t*‐statistic were obtained using threshold‐free cluster enhancement (TFCE).

Figure 7 shows the difference in between‐participants (second level) *t*‐statistics obtained without TFCE before and after MARSS in NYSPI‐GE and SBU‐Siemens task‐based data. Striped patterns in *t*‐statistic differences can be observed in both datasets, although these are masked by nonlinear distortions in the slice pattern of the artifact, which also differ across participants, due to preprocessing. *t*‐statistic maps reflecting the change in magnitude of *t*‐statistics can be seen in Figure S24 (if a negative *t*‐statistic became more negative, it would be shown as a decrease in Figure 7, but as an increase in Figure S24). Additionally, the difference in *t*‐statistics, as well as their magnitude difference, in between‐participants analyses performed with TFCE are shown in Figures 8 and S25, respectively. For all between‐participants analyses, the deciles of absolute value of *t*‐statistic change were calculated and are shown in Table 2. In NYSPI‐GE, 50% of *t*‐statistics changed by a magnitude of 0.171 or more (11.243 with TFCE), while in SBU‐Siemens 50% changed by a magnitude of 0.169 or more (3.041 with TFCE).

**TABLE 2:** Deciles of the greyordinate‐wise, absolute change in *t*‐statistic resulting from MARSS artifact correction in between‐participants analyses performed both with and without threshold‐free cluster enhancement (TFCE).

Deciles of absolute change in *t*‐statistic (“x% of T‐stats change by at least _”)										
Dataset	TFCE	90%	80%	70%	60%	50%	40%	30%	20%	10%
NYSPI‐GE	No TFCE	0.031	0.063	0.096	0.132	0.171	0.216	0.270	0.341	0.458
	TFCE	0.188	1.090	3.120	6.421	11.243	18.328	28.561	45.046	83.804
SBU‐Siemens	No TFCE	0.030	0.062	0.094	0.129	0.169	0.217	0.275	0.353	0.490
	TFCE	0.101	0.420	1.015	1.893	3.041	4.747	7.415	12.175	23.532

Abbreviations: NYSPI = New York State Psychiatric Institute; SBU = Stony Brook University; TFCE = threshold‐free cluster enhancement.

In NYSPI‐GE, the number of significant greyordinates increased from 28,659 to 28,918 (from 40,464 to 41,458 with TFCE) as a result of MARSS correction, with 1952 greyordinates (3138 with TFCE) becoming significant and 1693 (2144 with TFCE) losing significance. In SBU‐Siemens, the number of significant greyordinates increased from 8915 to 9828 (from 9564 to 10,936 with TFCE), with 1972 greyordinates (2039 with TFCE) becoming significant and 1059 (667 with TFCE) losing significance. Sørensen–Dice coefficients were 0.937 (0.936 with TFCE) and 0.838 (0.868 with TFCE) in NYSPI‐GE and SBU‐Siemens, respectively.

Within the left DLPFC ROI (Figure S1), the mean *t*‐statistic increased from 5.325 to 5.376 (+0.958%) without TFCE and from 1172.00 to 1240.00 (+5.80%) with TFCE in NYSPI‐GE. In SBU‐Siemens, the mean left DLPFC *t*‐statistic increased from 2.440 to 2.720 (+11.44%) without TFCE and from 68.231 to 83.512 (+22.40%) with TFCE.

## 4 Discussion

The results presented here demonstrate the successful mitigation of a shared artifactual signal between simultaneously acquired slices via MARSS correction. We have provided evidence that signal removed via MARSS is likely nonneural in nature, but that it impacts BOLD signal and task betas in gray matter. MARSS‐corrected data exhibits higher overall tSNR and lower cortical coefficients of variation than uncorrected data. Additionally, MARSS correction mitigates residual elevations in correlations between simultaneously acquired slices after denoising via sICA+FIX, RETROICOR, and slice‐timing correction. In task‐based analyses, MARSS results in decreased participant‐level task model error and removes spatial patterns of task betas at spatial frequencies corresponding to the distance between simultaneously acquired slices. Between participants, MARSS causes substantive changes in group‐level *t*‐statistics that tend to follow striped patterns along the slice acquisition plane, even after spatial normalization is performed. These results collectively indicate significant improvements in data quality following MARSS correction.

### 4.1 MARSS Correction Reduces Simultaneous Slice Correlations

We have shown the presence of a shared artifactual signal between simultaneously acquired slices of multiband fMRI data acquired across six datasets, including in two widely used publicly available datasets (Casey et al. 2018; Van Essen et al. 2013). This signal was originally identified as elevated correlations between timeseries in simultaneously acquired slices relative to adjacent‐to‐simultaneous slices, in both phantom and in vivo data, with elevations exceeding a difference in Pearson *r* correlation coefficients of 0.3 or 0.4 in many datasets. Further, the spatial pattern of elevated correlations follows a clear, systematic relationship with the MB factor used during acquisition. As there is no physiological justification for BOLD signal in arbitrary axial slices to be more related in simultaneously acquired slices than in any other given pair of slices (including neighboring slices), we developed a regression‐based detection and correction technique, MARSS, that estimates the shared signal across simultaneously acquired slices in unprocessed MB‐fMRI data and removes the estimated signal from each voxel in an fMRI run. This technique successfully reduces the magnitude of the difference in correlations between simultaneously acquired and adjacent‐to‐simultaneous slices.

Although MARSS also reduced simultaneous slice correlations to levels slightly below that of the average correlation between adjacent‐to‐simultaneous slices, it does so to a much lesser degree than the magnitude of the original elevated correlations. As shown in Table S3, the maximum negative difference in correlation between simultaneously acquired slices and adjacent‐to‐simultaneous slices induced by MARSS correction in any in vivo dataset is −0.085, as opposed to 0.465 in uncorrected data. Further, we observe that correlations between simultaneously acquired slices are not reduced below that of adjacent‐to‐simultaneous slices in gray matter, white matter, or CSF alone (see Figures S19 and S20), suggesting that the negative differences observed in correlation matrices across the entire image may not affect brain structures.

As an alternative to standard MARSS to address the negative differences between simultaneously acquired slices and adjacent‐to‐simultaneous slices, we also introduced MARSS30, MARSS20, and MARSS10, techniques that first obtain an average spatial distribution of isolated artifact signal using standard MARSS, then use the top 30%, 20%, or 10% of voxels in artifact magnitude, respectively, to estimate and remove the artifact signal. MARSS30 and MARSS20 produce smaller correlation differences between simultaneously acquired slices and adjacent‐to‐simultaneous slices (see Figure 2 and Table S6), but also do not reduce the elevated correlations between simultaneously acquired slices to the same extent as standard MARSS correction. This may point to a globally reduced effect of MARSS correction that further reduces when less and less voxels are used for artifact estimation. Nonetheless, these methods produce comparable results in the correction of PSDs of task betas (see Figure S23) to standard MARSS correction. As the 30%, 20%, and 10% artifact signal intensity thresholds were chosen based on the number of voxels per slice across the entire NYSPI‐GE task‐based dataset that would be included in the artifact estimation at each threshold, future work should be aimed at further threshold optimization across multiple datasets.

Although we collected only minimal data at lower MB factors (e.g., 3 or 4), the data in Table S4 raise the possibility that lower MB factors are associated with smaller magnitude reductions in simultaneous‐slice correlations after MARSS correction. This is consistent with the fact that lower MB factors should, a priori, produce poorer estimates of the shared signal across slices due to having fewer slices to average together to arrive at an estimate of the artifact.

It is important to acknowledge that utilizing slice correlation matrices to assess structured noise signal in unprocessed MB fMRI data is not a widely used technique. Thus, there are various correlation structures that can be observed, aside from those seen in simultaneously acquired slices due to MARSS artifact, whose origins are not fully understood. For example, structured correlations could be a result of residual in‐scanner head motion that was not fully modeled by 24 motion parameters, physiological motion, or magnetic field inhomogeneities or eddy currents during acquisition. These effects will vary on a run‐to‐run basis (see Figure S2 for single‐run examples), and thus the calculation of average slice correlation matrices is the most robust way to assess these structured correlations across an entire dataset. One example that can be seen within this manuscript are the clusters of high correlations that tend to exist between slices 20 and 60 in the NYSPI‐GE, SBU‐Siemens, and HCP‐Siemens datasets. These elevated correlations are likely due to shared global signal fluctuations across in‐brain voxels. A second example is the checkerboard pattern seen in all datasets (although most strongly in the SBU‐Siemens dataset; see Figures 1 and S3), which corresponds to the slice acquisition pattern and is corrected via STC (see Figure S8).

### 4.2 MARSS Correction Improves SNR and Removes Nonneural Signal

Critically, we show that MARSS artifact correction results in the removal of image noise rather than true neural signal. First, an increase in mean, whole‐brain tSNR in 100% of unprocessed runs across all datasets after MARSS correction, which is more pronounced in gray matter than white matter (see Table S10), suggests improved data quality. In preprocessed data, the largest mean increases in tSNR occurred in cortical regions, while many decreases occurred at tissue boundaries where BOLD signal most likely contained greater contamination from fluid motion or vascular signal (Figures S15–S18). Although tSNR carries no information on activation strength in task‐based fMRI data, it is a useful measure of signal stability in studies of resting‐state fMRI (De Blasi et al. 2020).

One major indication that MARSS predominantly removes nonneural signal lies in the spatial distribution of the estimated artifact in Figure 3, where the largest amount of signal is removed from large vasculature, and a higher percentage of mean signal is removed from the background of the image. The significant vascular involvement suggests that artifact correction is isolating signal related to vasomotion or pulsatile blood flow whose contribution elevates signal correlations between simultaneously acquired slices. It is plausible that this signal originates from systemic low‐frequency oscillations (sLFOs), which are considered nonneural in nature and account for a significant portion of the variance in signal in the range of approximately 0.01–0.15 Hz (Frederick, Nickerson, and Tong 2012; Tong, Hocke, and Frederick 2019). Notably, isolated artifact timeseries show autocorrelations up to lags that approximately correspond with this frequency range (Figures S10 and S13).

The frequency band occupied by sLFOs is the same as that which is occupied by fluctuations in neural activity that are the basis of resting‐state functional connectivity. Thus, traditional spectral filtering techniques cannot remove these signals without compromising true signal from neuronal fluctuations. Tong et al. (2015) demonstrated that sLFOs can confound estimates of resting‐state functional connectivity, going as far as mimicking resting‐state networks isolated via independent components analysis. Additionally, they have identified major vascular fMRI signal components that appear in the spatial distribution of the artifact signal, such as the superior sagittal sinus, to be highly correlated with simultaneously‐acquired near infrared spectroscopy estimates of blood flow (Tong et al. 2013).

Slightly complicating this explanation of the artifact source is its presence in phantom data. The artifact signal intensity in phantom data is strongest near the point of contact with the scanner table (see Figure S9). Due to this being a liquid‐filled phantom, it is possible that scanner table vibrations caused periodic fluid motion that resembles pulsatile blood flow. To this point, any time‐varying noise source, such as vascular pulsation, blood flow, or head motion (which we demonstrate is associated with artifact magnitude in Figure S14), whose phase is independent of acquisition timing may be captured as shared signal between simultaneously acquired slices. For example, the artifact may originate from the transit of perturbed proton spins in a periodic fashion through fluid‐filled spaces, such as major neurovasculature and the eyes. While currently inconclusive, the true source of the MARSS artifact bears further investigation.

Given that MARSS estimates and corrects for a structured noise signal that relies on the simultaneous slice acquisition pattern in unprocessed scanner space for identification, denoising techniques such as sICA+FIX that are typically performed on data that has been normalized to a template space, which distorts the slice acquisition pattern, would not be expected to fully account for this signal. This is evident in the correlation matrices of resting‐state data from the HCP‐Siemens dataset that has been denoised using sICA+FIX and decomposed into neural and random noise subspaces (see Figure 5). Prior to MARSS correction, elevated correlations between simultaneously acquired slices show up strongly in both subspaces, indicating that a portion of the artifact signal was likely modeled as true neural signal across multiple ICs, and the remaining artifact signal in the cleaned data was not modeled. Further, GSR is not able to correct the elevated correlations in the neural subspace, instead slightly exacerbating them. In contrast, MARSS correction prior to sICA+FIX denoising significantly reduces artifact presence across all subspaces, resulting in statistically insignificant correlation differences between simultaneously acquired slices and adjacent‐to‐simultaneous slices. Correlation matrices of the difference between uncorrected and MARSS‐corrected data do not exhibit any structured correlation patterns besides that between simultaneously acquired slices, indicating that MARSS correction is most likely not removing neural signal from any subspace. While further evaluation of sICA+FIX denoising in MARSS‐corrected fMRI data is needed, these results suggest that MARSS and sICA+FIX denoising used in tandem may provide maximal denoising of structured artifact signals.

### 4.3 MARSS Correction Reduces Cortical Coefficient of Variation

The removal of noise from unprocessed, volumetric data due to artifact correction is beneficial to volume‐to‐surface mapping in the HCP MPP (Glasser et al. 2013). For context, the mapping algorithm excludes voxels with coefficients of variation that are 0.5 standard deviations above the mean local coefficient of variation in a 5 mm Gaussian neighborhood. Both the NYSPI‐GE and SBU‐Siemens datasets experience decreases in coefficient of variation in 97.00% and 80.77% of voxels, respectively. This nearly global decrease suggests an increase in probability of most cortical voxels to be included in volume to surface mapping. Notably, several runs of NYSPI‐GE data were discovered to have striped patterns of high coefficients of variation (see Figure S26) that are mitigated after MARSS correction. Upon inspection, data from these runs show no discernable quality control concerns, suggesting that multiband acquisition can sometimes cause spurious, yet systematic noise patterns that could potentially compromise surface timeseries extraction in otherwise usable data.

### 4.4 MARSS Correction Improves Spatial Statistics Within and Between Participants

Critically, we have shown that MARSS correction has substantial implications for spatial statistics in task‐based analyses, both within and between participants. When comparing within‐participants analyses in unprocessed data prior to and following MARSS correction, we observe a striping pattern in task beta differences across all task‐based datasets (Figure 6, top row). We analyzed the spatial frequency of these patterns along the slice direction and demonstrated that systematic patterns in task betas occur at the fundamental frequency and harmonic frequencies of the simultaneously acquired slice pattern in uncorrected data (Figure 6, bottom two rows), and that the spectral power at these spatial frequencies is reduced by MARSS. While there is no ground truth expectation as to the magnitude of task betas, it is reasonable to conclude that true estimates of task‐evoked neuronal activation should not exhibit a spatial frequency dependent on the spacing between simultaneously acquired slices. The fact that MARSS correction reduces the spectral power of these patterns indicates that this spatial frequency is being removed from the data by our method, rather than being added.

In within‐participants analyses of preprocessed, task‐based data, we observe statistically significant decreases in mean‐squared residuals, indicating lower model error and suggesting that task‐irrelevant, and potentially nonneural, signals are being removed by MARSS. Additionally, we observe decreases in BIC for both the NYSPI‐GE and SBU‐Siemens datasets following MARSS, even while accounting for the loss of a degree of freedom at the step of removing the MARSS artifact signal. At the group level, we observe striping patterns in the difference in *t*‐statistics between analyses performed before and after MARSS correction (Figures 7 and 8). Spatial frequency analyses demonstrated that the systematic pattern is present first in unprocessed, uncorrected data and persists in the data even after spatial normalization and volume‐to‐surface resampling distort the slice acquisition pattern to varying degrees in each individual participant. MARSS correction led to substantial changes in between‐participants *t*‐statistics, with 10% of greyordinates changing by at least 0.458 and 0.490 in NYSPI‐GE and SBU‐Siemens, respectively (Table 2). This resulted in a net increase in the number of significant greyordinates, both with and without the use of TFCE. Sørensen–Dice coefficients for each dataset indicate a change in significance in approximately 6.63% (6.64% with TFCE) greyordinates in NYSPI‐GE and 16.20% (13.20% with TFCE) greyordinates in SBU‐Siemens. While high Sørensen–Dice coefficients are typically desirable in the validation of image segmentation algorithms and other deep learning applications, a lower coefficient in this application indicates a greater change in spatial statistics induced by MARSS correction.

Since there is no ground truth expectation for estimations of whole‐brain, task‐evoked activation, we then constrained our analysis to within the left DLPFC, which is known to show robust activation in response to working memory task demands (Barbey, Koenigs, and Grafman 2013; Funahashi, Takeda, and Watanabe 2004; Hampson et al. 2013). Within the chosen ROI, which was derived from previous group‐level analysis of the SOT in a separate dataset (Van Snellenberg et al. 2016, 2015), *t*‐statistics increase in both NYSPI‐GE and SBU‐Siemens with and without the use of TFCE. The larger percentage increase in *t*‐statistic that results from using TFCE suggests that removal of noise due to artifact correction enhances the spatial homogeneity of patterns of true task‐evoked activation, leading to clearer identification and amplification of significant regions by the TFCE algorithm.

### 4.5 Differences in Results Between Datasets

One theme in the results presented here is that NYSPI‐GE appears to show less improvement than SBU‐Siemens in between‐participants analyses following MARSS correction. There are several hardware differences between these datasets that may influence this, such as scanner manufacturer and coil configuration. Further, the slightly longer TR used in NYSPI‐GE data could result in diminished temporal resolution and less adequate sampling of artifactual periodic signals identified by artifact estimation.

If not solely due to hardware and acquisition differences, it is likely that the smaller sample size of the SBU‐Siemens dataset results in a larger observed change in between‐participants results than in the NYSPI‐GE dataset. Since changes to spatial patterns of task activation that occur due to MARSS correction will vary across individuals based on head position in the scanner, as well as differences in brain size and shape, differences between individuals should “average out” in between‐participants results with a large enough sample size. However, if this is the case, it would also be the case that individual‐difference analyses, such as correlations of single‐subject brain activation or connectivity patterns with clinical or cognitive measures (such as symptom measures in a patient population, or neuropsychological or cognitive task performance) could be heavily impacted by MARSS correction, as individual‐subject maps would not be subjected to any averaging that would reduce the impact of MARSS correction on results.

Despite the observed differences between these datasets, the spatial distribution of isolated artifact signal (Figure 3) in NYSPI‐GE is highly similar to that of SBU‐Siemens; differences in smoothness of the spatial distribution can be attributed to the smaller sample size of SBU‐Siemens (*N* = 10) relative to that of NYSPI‐GE (*N* = 63). Additionally, both datasets experience improvements in tSNR and reductions in cortical COV, as well as reduction of artificially elevated PSD of task betas at fundamental and harmonic frequencies related to the simultaneous slice acquisition, indicating improvement in task modeling regardless of the resulting change in between‐participants *t*‐statistics.

In the NYSPI‐GE, SBU‐Siemens, and HCP‐Siemens datasets, there are visual differences between spatial PSD estimates of task betas (Figure 6) across datasets, as well as between the task and control conditions. Particularly, PSDs of task and control condition betas in uncorrected NYSPI‐GE and SBU‐Siemens data look markedly different within each dataset, as well as across datasets. This could be attributed to acquisition differences described above. Within datasets, there are notably fewer control trials than task condition trials, potentially resulting in a less stable estimation of control condition betas. Further, the HCP‐Siemens dataset seems to experience less correction than the other two datasets at the harmonic spatial frequencies, again potentially due to acquisition differences (e.g., faster TR, stronger gradients, differences in phase‐encode direction, or differences in the magnitude of task‐evoked activation by the dataset's working memory task). Interestingly, the harmonic frequency that experiences the least correction following MARSS is present in signal isolated via RETROICOR performed both with and without MARSS (see Figure S22), suggesting that RETROICOR and MARSS in tandem may provide maximal correction of systematic task beta patterns. Overall, all PSDs within and across scanners look more similar to each other following MARSS correction.

### 4.6 Limitations and Future Considerations

This study establishes the foundation for several analyses to further investigate the source of the observed artifact signal that have yet to be conducted. First, the true impact of scanner manufacturer or platform would best be investigated using an identical phantom, and potentially human participants, and acquisition sequence across multiple scanners. Within this analysis, scanner vibration (Berl et al. 2015) should be recorded to assess the impact of mechanical noise on artifact magnitude. Additionally, as we do not rule out head motion as a potential artifact source, a more thorough investigation of the potential role of motion in contributing to the artifact should be performed. Ideally, this should include the estimation of moment‐to‐moment motion for each simultaneous slice set using continuous motion tracking via an in‐scanner laser or camera apparatus, as motion effects are most likely incompletely modeled by volume‐wise motion parameters. Next, acquisition parameters such as partial Fourier sampling factor and phase‐encode direction should be varied in order to determine if there are optimal combinations of parameters that minimize correlation elevations between simultaneously acquired slices. The effect of variations in raw data reconstruction pipeline should also be assessed, including an evaluation of artifact presence in data reconstructed using slice‐GRAPPA (Setsompop et al. 2011) and split‐slice GRAPPA (Cauley et al. 2014).

While we have demonstrated that a shared artifact signal between simultaneously acquired slices is present even after denoising via sICA+FIX and RETROICOR, subsequent analyses should include an evaluation of MARSS correction used both in tandem with and against other existing denoising techniques, such as temporal ICA (Glasser et al. 2018, 2019), ICA AROMA (Pruim et al. 2015), and ANATICOR (Jo et al. 2020, 2010).

Furthermore, the sICA+FIX denoising analyses presented here used ICs estimated on uncorrected data, but then applied to MARSS‐corrected data. A full examination of how MARSS and sICA+FIX or other ICA‐based methods interact would require the estimation of ICA components on MARSS‐corrected data. These analyses should include a comparison of both estimates of task‐evoked activation and resting‐state functional connectivity across techniques.

An additional limitation is that our examination of MARSS denoising here is primarily restricted to MB acceleration factors of 6 and 8. As noted in Section 4.1 above, lower MB acceleration factors should be expected to produce poorer estimates of the artifact signal (due to the existence of fewer simultaneously‐acquired slices to estimate the artifact from), and there is no guarantee that our results would hold at lower MB factors. At a minimum, application of MARSS should probably not be conducted at an MB factor of 2, as this would effectively involve estimating the MARSS artifact from only a single slice, with no averaging of spatially disparate slices that helps to mitigate the impact of true underlying neural signals on the estimated artifact signal.

### 4.7 Conclusion

In summary, we have demonstrated the presence and successful correction of an artifactual, nonneural signal that is shared between simultaneously acquired slices in multiple datasets. Removal of this artifact with the MARSS method developed here results in substantive improvements in data quality, including nearly global increases in tSNR and improvements to the extraction of the cortical surface during preprocessing. The artifact appears strongest in the neurovasculature, but has clearly demonstrable impacts throughout gray matter voxels and greyordinates. Further, the artifact is not fully accounted for by sICA+FIX denoising, likely because ICA components are estimated after spatial distortions of the raw scanner space data have occurred during preprocessing, thereby mixing artifact signals from adjacent‐to‐simultaneous slices together and making the true original MARSS artifact signal unrecoverable following standard preprocessing. The artifact is also not completely accounted for by physiological correction via RETROICOR. In addition, MARSS correction leads to a reduction of systematic spatial patterns in estimates of task‐evoked activation related to slice acquisition patterns, both within and between participants, which ultimately leads to substantive changes in group‐level *t*‐statistics throughout the brain. We expect that application of our artifact correction method will lead to an improvement in both the quality and reproducibility of all multiband fMRI datasets, and recommend that investigators implement this technique prior to functional data preprocessing, at least for MB factors of 6 or higher.

## Conflicts of Interest

The authors declare no conflicts of interest.

## Supporting information


**Data S1.** Supporting Information.

**Video S1.** Three‐dimensional view of the average spatial distribution of isolated artifact signal in resting‐state data from NYSPI‐GE (*N* = 63).

## Data Availability

Data used in this manuscript can be made available by the corresponding author through a formal data sharing agreement upon request. Data were provided, in part, by the Human Connectome Project, WU‐Minn Consortium (Principal Investigators: David Van Essen and Kamil Ugurbil; U54MH091657) funded by the 16 NIH Institutes and Centers that support the NIH Blueprint for Neuroscience Research; and by the McDonnell Center for Systems Neuroscience at Washington University. HCP data can be obtained from ConnectomeDB (https://db.humanconnectome.org) (Hodge et al. 2016). Data used in the preparation of this article were obtained from the Adolescent Brain Cognitive Development (ABCD) Study (https://abcdstudy.org), held in the NIMH Data Archive (NDA). This is a multisite, longitudinal study designed to recruit more than 10,000 children age 9–10 and follow them over 10 years into early adulthood. The ABCD Study is supported by the National Institutes of Health and additional federal partners under award numbers U01DA041048, U01DA050989, U01DA051016, U01DA041022, U01DA051018, U01DA051037, U01DA050987, U01DA041174, U01DA041106, U01DA041117, U01DA041028, U01DA041134, U01DA050988, U01DA051039, U01DA041156, U01DA041025, U01DA041120, U01DA051038, U01DA041148, U01DA041093, U01DA041089, U24DA041123, U24DA041147. A full list of supporters is available at https://abcdstudy.org/federal‐partners.html. A listing of participating sites and a complete listing of the study investigators can be found at https://abcdstudy.org/consortium_members/. ABCD consortium investigators designed and implemented the study and/or provided data but did not necessarily participate in the analysis or writing of this report. This manuscript reflects the views of the authors and may not reflect the opinions or views of the NIH or ABCD consortium investigators. MARSS is available as an open‐source MATLAB software package on GitHub (https://github.com/CNaP‐Lab/MARSS) and the MathWorks File Exchange (https://www.mathworks.com/matlabcentral/fileexchange/156782‐multiband‐artifact‐regression‐in‐simultaneous‐slices‐marss). Code to replicate all analyses in this manuscript can be made available by the authors upon request.
