# Supplementary material for: Characterization and Mitigation of a Simultaneous Multi‐Slice fMRI Artifact: Multiband Artifact Regression in Simultaneous Slices
Source: Hum Brain Mapp. 2024 Nov 6;45(16):e70066. doi: 10.1002/hbm.70066 (PMC11538719; doi:10.1002/hbm.70066)
Supplement: Supplementary file 1 — Data S1. Supporting Information. [file HBM-45-e70066-s002.docx]

# **Supplemental Information**

**Characterization and Mitigation of a Simultaneous Multi-Slice fMRI Artifact: Multiband Artifact Regression in Simultaneous Slices**

Tubiolo *et al.*

**These supplementary materials include:**

Supplementary Methods

Supplementary Results and Discussion

Supplementary Figures S1 – S26

Supplementary Tables S1 – S13

Supplementary References

**Supplementary Methods**

**Frequency Spectral Analysis**

For each run, in each given dataset, the average, whole-image, isolated artifact timeseries was mean-centered. All runs were then concatenated to form a single vector, with zero-padding of the length of the timeseries included after each sample. Welch’s power spectral density (PSD; Welch, 1967) was calculated on the concatenated timeseries with a Hanning window applied to each individual timeseries, and 50% overlap between samples. These same procedures were utilized in all other frequency spectral analyses in the manuscript (see main text, *2.6.2 Spatial Frequency Analysis of Unprocessed Task fMRI*).

To quantify the average relationship between PSD and frequency in the roughly linear portion of the spectrum (with a lower frequency bound of -2 log[Hz]), a linear regression was performed on log-log transformed PSD that had been weighted by the inverse kernel density of the spectrum. The slope of this regression is useful in estimating colored noise characteristics of the artifact signal.

**Autocorrelation**

To investigate the potential periodicity of the isolated artifact signal, we calculated its autocorrelation as a function of lag, with each lag representing a temporal shift in the signal of one TR. In each dataset, the isolated artifact timeseries, obtained from Equation 11, above, was spatially averaged to produce a single mean artifact timeseries. This timeseries was then mean-centered and linearly detrended before calculating the autocorrelation up to 20 lags (or a shift of 20 TRs) to sufficiently account for the period of physiological signals that may be components of the artifact signal. Autocorrelations were then averaged across runs in each dataset.

**Associations between Artifact Magnitude and In-Scanner Motion**

We sought to determine whether the artifact magnitude, measured as the elevation in Pearson correlation in simultaneously acquired slices compared to adjacent-to-simultaneous slices, was associated with measures of in-scanner subject motion. These associations were assessed in resting-state and task-based data from the NYSPI-GE dataset. For each subject, the mean artifact magnitude was calculated as the average of the Fisher Z-transformed simultaneously acquired slice correlation elevations across runs. In-scanner motion was quantified either by 1) framewise displacement (FD), calculated as the sum of the absolute values of the derivative of six motion parameters (X, Y, Z, roll, pitch, and yaw), and 2) DVARS (temporal derivative root-mean-squared variance over voxels), calculated as the root-mean-square of the derivative of the timeseries across all in-brain voxels (J. D. Power, Barnes, Snyder, Schlaggar, & Petersen, 2012; Jonathan D. Power et al., 2014; Smyser et al., 2010). The mean and median of each motion measure were calculated across runs for each subject, and separate robust linear regressions were used to predict artifact magnitude from each mean and median motion measure.

**Effect of Slice Timing Correction on Artifact Magnitude**

In order to assess the effect of performing slice timing correction (STC) on artifact presence and magnitude, we performed STC on the unprocessed, resting-state, SBU-Siemens data using SPM12 (Friston, 2007; Friston et al., 2004). Artifact presence was then evaluated using prior methods (see main text, *2.2* *Identification of Artifact Signal in Simultaneously Acquired Slices*). Additionally, STC was performed in both single-band datasets; slice correlation matrices were calculated prior to and following STC.

**Evaluation of MARSS Correction in Gray Matter, White Matter, and Cerebrospinal Fluid**

We sought to quantify the comparative effects of MARSS correction in gray matter, white matter, and cerebrospinal fluid (CSF). In the NYSPI-GE resting-state and task-based datasets, tissue segmentations for each run were obtained as preprocessing outputs (*aparc+aseg.nii.gz*; see main text, *2.6.3 fMRI Preprocessing*), and warped into the unprocessed subject space using *-volume-resample* in Connectome Workbench Command v2.0.0 (Marcus et al., 2011; Van Essen et al., 2011). Artifact presence in uncorrected and MARSS-corrected data (see main text, *2.2* *Identification of Artifact Signal in Simultaneously Acquired Slices*), mean artifact signal magnitude, and change in tSNR (see main text, *2.4.7* *Temporal Signal-to-Noise Ratio*) were then quantified separately in gray matter, white matter, and CSF.

**Task Procedures**

*Self-ordered Working Memory Task (SOT)*

The Self-ordered Working Memory Task (SOT) was modified from previously published work (Van Snellenberg, Conway, Spicer, Read, & Smith, 2014; Van Snellenberg et al., 2016; Van Snellenberg et al., 2015), and consisted of 20 trials, with eight steps of gradually increasing working memory (WM) load in each trial. At the start of a trial, a three-by-three grid of eight simple line drawings of three-dimensional objects was presented, with the center position in the grid left blank. Unique stimuli were used on each of the first 10 trials (80 total stimuli) and were each repeated once in the following 10 trials. Participants were given seven seconds in which to respond on each step. Responses consisted of using an fMRI compatible trackball to position a cursor over one of the objects and select it with a button press. Participants were instructed to select any object on each step that they had not already selected on a previous step (thus, on the first step all possible responses are correct). Once participants made a selection, a white square was displayed around the selected object until a total of nine seconds had elapsed since the beginning of the step, thus ensuring that each step remained the same length regardless of participants’ reaction times. At the start of each subsequent step after the first, the objects were pseudo-randomly rearranged in the grid, but with the blank space placed at the location of the previously selected item (thus preventing participants from simply selecting the same location on each step). If participants failed to make a response within seven seconds from the beginning of a step, a white square was displayed around a randomly selected object that would have been a correct response. Participants were instructed to remember this object as if they had selected it themselves, and to continue the trial. If an incorrect selection was made, a red square was displayed over top of the selected object in order to indicate that an error had been made, and the same procedure as in the case of no response was followed. Finally, participants also carried out two trials of a control task, in which one of the objects on each step was marked with an asterisk and participants were instructed to simply select the marked object. In all other respects the display and randomization of stimuli for the control task was identical to the SOT. Unique stimuli were used for each of the control trials, and each trial of either type was preceded by textual instructions indicating whether the upcoming trial was a task trial or a control trial.

**Variance Decomposition of fMRI Data Subspaces**

In addition to performing a subspace decomposition of sICA+FIX denoised data, we sought to characterize the effect of MARSS correction on the distribution of variance between these subspaces. The full description and rationale of this decomposition is described in detail elsewhere (see Supplementary Materials in Glasser et al., 2018). Briefly, the decomposition of variance in raw, unprocessed data $Var_{Raw}$ can be written as:

$$\begin{aligned} Var_{Raw}\boldsymbol{=Va}\boldsymbol{r}_{\boldsymbol{Detrend}}\boldsymbol{+Va}\boldsymbol{r}_{\boldsymbol{MP}\boldsymbol{24}}\boldsymbol{+Va}\boldsymbol{r}_{\boldsymbol{NoiseICA}}\boldsymbol{+Va}\boldsymbol{r}_{\boldsymbol{Neural}}\boldsymbol{+Va}\boldsymbol{r}_{\boldsymbol{Random}}\boldsymbol{\#}\left( 1 \right) \end{aligned}$$

where $Var_{Detrend}$ is calculated from signal removed via linear detrending, $Var_{MP24}$ is calculated from signal removed by regression of six motion parameters, their squares, derivatives, and squared derivatives, $Var_{NoiseICA}$ is the variance estimated from noise independent components, $Var_{Neural}$ is the variance of the neural signal subspace, and $Var_{Random}$ is the variance of the random noise subspace. With the addition of MARSS correction, a new term $Var_{MARSS}$ is introduced such that $Var_{Raw}$ can now be expressed as

$$\begin{aligned} Var_{Raw}\boldsymbol{=Va}\boldsymbol{r}_{\boldsymbol{Detrend}}\boldsymbol{+Va}\boldsymbol{r}_{\boldsymbol{MP}\boldsymbol{24}}\boldsymbol{+Va}\boldsymbol{r}_{\boldsymbol{NoiseICA}}\boldsymbol{+Va}\boldsymbol{r}_{\boldsymbol{Neural}}\boldsymbol{+Va}\boldsymbol{r}_{\boldsymbol{Random}}\boldsymbol{+Va}\boldsymbol{r}_{\boldsymbol{MARSS}}\boldsymbol{\#}\left( 2 \right) \end{aligned}$$

Methods for calculating each subspace are described elsewhere (see main text, *2.5.2. Subspace Decomposition of sICA+FIX Denoised Data*). For each resting-state run of each participant in the HCP-Siemens dataset, the variance terms in the above equations were calculated in uncorrected and MARSS-corrected data, the only difference being that $Var_{MARSS}$ cannot be calculated for uncorrected data. Variance for each subspace was calculated as the mean variance of all in-brain voxels.

We were also particularly interested in calculating the percentage of variance explained by the MARSS artifact that was removed from the neural signal subspace as a measure of the magnitude of artifact signal that was incorrectly modeled as true neural signal by sICA+FIX. This was done by calculating the variance of the difference between the uncorrected neural signal subspace and the MARSS-corrected neural signal subspace and dividing by the variance of the MARSS-corrected neural signal subspace.

**Supplemtary Results and Discussion**

Variance Decomposition of fMRI Data Subspaces

The results of a variance decomposition of data subspaces in both uncorrected and MARSS-corrected resting-state data from the HCP-Siemens dataset are shown in **Table S13**. Decreases in mean voxel-wise variance were observed in the signal removed by linear detrending, motion parameter regression, and sICA+FIX noise components, while increases in variance were observed in the neural signal subspace and random noise subspace. Standard error of the mean variance slightly decreased in all subspaces. The variance of the difference between the uncorrected and MARSS-corrected neural subspaces was calculated to be 67.857 ± 10.490 (confidence interval reflect 2 * standard error of the mean). Consequently, signal changes in the neural subspace by MARSS correction explains approximately 1.128% of the variance in the neural subspace.

Across all subspaces, the largest decrease in variance was observed in the sICA+FIX noise component subspace. This may indicate that MARSS correction removes a portion of the structured noise that would have been estimated and removed by sICA+FIX noise components. As a result of the reduced signal magnitude being removed by these components, a slightly higher signal variance is observed in the neural signal subspace, likely suggesting the return of true variance to this subspace that had been previously estimated as noise, as well as an increase in the variance of unstructured noise in the random noise subspace.

While these results indicate an overall improvement in both the performance of sICA+FIX and in data quality, this analysis is limited by the fact that the independent component regressors used to performed sICA+FIX denoising were previously estimated on data that had been normalized to a template space (and subsequently applied to unprocessed data in the scanner space). This normalization distorts the simultaneous slice acquisition pattern, and thus sICA+FIX is not expected to be able to identify noise signals following that structure. Additionally, independent component regressors that were estimated on uncorrected data were used on MARSS-corrected data. Therefore, this analysis should be repeated in future work with the added step of estimating sICA+FIX components on MARSS-corrected data, specifically.

**Supplementary Figures**

**Figure S1.** Left dorsolateral prefrontal cortex region of interest used for evaluation of changes in group-level t-statistics due to artifact correction.

**Figure S2.** Pearson correlation matrices between average signal in all slice pairs in A) unprocessed data and B) MARSS-artifact corrected data (all N = 1), performed in phantom and in vivo at varying multiband (MB) acceleration factors.

**Figure S3.** Pearson correlation matrices between average signal in all slice pairs in unprocessed and MARSS-corrected resting-state data from NYSPI-GE (N = 63), SBU-Siemens (N = 10), HCP-Siemens (N = 25), ABCD-Siemens (N = 25), ABCD-GE (N = 3) and ABCD-Philips (N = 7).

**Figure S4.** Pearson correlation matrices between average signal in all slice pairs in the NYSPI-Philips Self-Ordered Working Memory Task (SOT; N = 27; A, E) and N-Back (N = 22; B, F) datasets, as well as the QTIM-Bruker Resting-state (N = 46; C, G) and N-Back (N = 46; D, H) datasets. Both datasets were collected using single-band acquisition. Correlation matrices were calculated before and after slice-timing correction (bottom row).

**Figure S5.** Pearson correlation matrices between average signal in all slice pairs in uncorrected data and data corrected with the alternative technique of estimating artifact signal from image background alone. Analysis was performed in resting-state data from SBU-Siemens (N = 10).

**Figure S6.** Pearson correlation matrices between average signal in all slice pairs in uncorrected data and data corrected by slice leakage regression. Analysis was performed in resting-state data from SBU-Siemens (N = 10).

**Figure S7.** Average signal correlations between voxels in simultaneously acquired slices as a function of the phase shift between them during multiband reconstruction in resting-state data from the SBU-Siemens dataset (N = 10). The phase shift in this dataset was 34 voxels (marked with grey dashed line), meaning that voxels in a given slice overlapped with the voxels that were exactly 34 voxels away along the phase-encode direction during reconstruction. Analysis was performed in uncorrected data (blue line), MARSS-corrected data (red line), and uncorrected data that had undergone slice leakage regression, only (green line).

**Figure S8.** Pearson correlation matrices between average signal in all slice pairs in uncorrected data and data that has undergone slice timing correction. Analysis was performed in resting-state data from SBU-Siemens (N = 10).

**Figure S9.** Average spatial distribution of the isolated artifact signal in SBU-Siemens phantom data collected with a multiband factor of 6 (N = 1). The black portion of the image occurred to an “amplifier blocking” effect in which signal intensity reached the maximum value in those voxels, resulting on a variance of 0, preventing the artifact from being estimated in those voxels.

**Figure S10.** Log-log transformed power spectral density (PSD) of the isolated artifact timeseries concatenated across participants (top row) and average autocorrelation (bottom row) in task-based fMRI data from NYSPI-GE (N = 58; A,D), SBU-Siemens (N = 10; B,E), and HCP-Siemens (N = 25; C,F). PSD plot annotations denote the slope (β) and R^2^ of each linear fit (red line). Grey areas with dotted borders in autocorrelation plots denote the 95% confidence interval for significant autocorrelation over and above that of white noise.

**Figure S11.** Log-log transformed power spectral density of the isolated artifact timeseries concatenated across all participants in resting-state fMRI data from A) NYSPI-GE (N = 63), B) SBU-Siemens (N = 10), C) HCP-Siemens (N = 25), D) ABCD-Siemens (N = 25), E) ABCD-GE (N = 3), and F) ABCD-Philips (N = 7). Plot annotation denotes the slope (β) and R^2^ of each linear fit (red line).

**Figure S12.** Log-log transformed power spectral density of the isolated artifact timeseries in SBU-Siemens phantom data collected with a multiband factor of 6 (N = 1). Plot annotation denotes the slope (β) and R^2^ of the linear fit (red line).

**Figure S13.** Mean, Z-transformed autocorrelation of the isolated artifact timeseries as a function of lag in resting-state fMRI data from A) NYSPI-GE (N = 63), B) SBU-Siemens (N = 10), C) HCP-Siemens (N = 25), D) ABCD-Siemens (N = 25), E) ABCD-GE (N = 3), and F) ABCD-Philips (N = 7). Grey area with dotted borders denotes the 95% confidence interval for significant autocorrelation over and above that of white noise.

**Figure S14.** Associations between artifact magnitude, measured as the difference in Z-transformed Pearson correlation between simultaneously acquired slices and adjacent slices, and mean framewise displacement (FD; A, C), median FD (B, D), mean DV (E, G), and median DV (F, H). Inlaid text denotes slope (β) and p-value (p) of linear fit, with red text denoting statistical significance at p<0.05. Analyses were performed in the NYSPI-GE resting-state (N = 63) and task-based (N = 58) datasets.

**Figure S15.** Mean, voxel-wise change in temporal signal-to-noise ratio (tSNR; A,B) and Coefficient of Variation (COV, C,D) as a result of MARSS artifact correction in preprocessed, task-based fMRI data from NYSPI-GE (N = 58; A,C) and SBU-Siemens (N = 10; B,D).

**Figure S16.** Mean, voxel-wise percent change in temporal signal-to-noise ratio (tSNR; A,B) and Coefficient of Variation (COV, C,D) as a result of MARSS artifact correction in preprocessed, task-based fMRI data from NYSPI-GE (N = 58; A,C) and SBU-Siemens (N = 10; B,D).

**Figure S17.** Mean, voxel-wise change in temporal signal-to-noise ratio as a result of MARSS artifact correction in preprocessed, resting-state fMRI data from A) NYSPI-GE (N = 63) and B) SBU-Siemens (N = 10).

**Figure S18.** Mean, voxel-wise percent change in temporal signal-to-noise ratio as a result of MARSS artifact correction in preprocessed, resting-state fMRI data from A) NYSPI-GE (N = 63) and B) SBU-Siemens (N = 10).

**Figure S19.** Pearson correlation matrices between average signal in all slice pairs in the NYSPI-GE task-based dataset (N = 58) prior to and following MARSS correction, calculated using only voxels containing gray matter, white matter, and cerebrospinal fluid, respectively. Correlation matrices only show slices in which all runs of all participants contained voxels of each tissue type.

**Figure S20.** Pearson correlation matrices between average signal in all slice pairs in the NYSPI-GE resting-state dataset (N = 63) prior to and following MARSS correction, calculated using only voxels containing gray matter, white matter, and cerebrospinal fluid, respectively. Correlation matrices only show slices in which all runs of all participants contained voxels of each tissue type.

**Figure S21.** Mean spatial distributions of signal removed via MARSS correction alone (A) and via RETROICOR alone (B) in the HCP-Siemens resting-state dataset (N = 25).

**Figure S22.** Power spectral density of mean spatial distributions of signal removed by RETROICOR alone (blue line) and by RETROICOR following MARSS correction (red line) in the HCP-Siemens resting-state dataset (N = 25). Vertical lines denote the fundamental spatial frequency (solid grey line) and harmonic spatial frequencies (dotted grey lines) associated with the simultaneous slice pattern.

**Figure S23.** Power spectral density (PSD) of average within-participants betas as a function of spatial frequency in NYSPI-GE uncorrected data (blue line), MARSS-corrected data (red line), and data corrected with MARSS procedures with artifact signal intensity thresholds (MARSS30, MARSS20, and MARSS10; orange, green, and purple lines, respectively). Vertical lines denote the fundamental spatial frequency (solid grey line) and harmonic spatial frequencies (dotted grey lines) associated with the simultaneous slice pattern. PSD is calculated separately for the task-on condition (A) and control condition (B) of the task.

**Figure S24.** Change in t-statistic magnitude in between-participants analysis of task-evoked activation as a result of MARSS artifact correction in data from A) NYSPI-GE (N = 52) and B) SBU-Siemens (N = 9).

**Figure S25.** Change in t-statistic magnitude in between-participants analysis of task-evoked activation as a result of MARSS artifact correction in data collected from A) NYSPI-GE (N = 52) and B) SBU-Siemens (N = 9). T-statistic maps before and after correction were generated using threshold-free cluster enhancement (TFCE) during permutation testing.

**Figure S26.** Cortical coefficient of variance (COV) before and after MARSS artifact correction in a task-based fMRI run from a single participant in the NYSPI-GE dataset (N = 1). Clear stripes of high COV in uncorrected data resulted in the exclusion of these voxels from this participant’s volume-to-surface mapping process.

**Supplementary Tables**

| **Dataset** | **N** | **Mean Age [SD]** | **M/F** | **Race** | **Ethnicity** |
| --- | --- | --- | --- | --- | --- |
| SBU | 10 | 28 [9.43] | 10/0 | 7W/1AA/0AS/2 Other | 2H/8NH |
| NYSPI | 66 | 32 [11.13] | 48/18 | 17W/31AA/6AS/4MR/8 Other/Unknown | 17H/49NH |
| HCP | 25 | 29.08 [3.73] | 18/7 | 18W/6AA/1AS | 24NH/1 Unknown |
| ABCD | 35 | 10.29 [1.16] | 18/17 | 20W/5AA/2AS/1MR/1 Other/3 Unknown | 12H/20NH/3 Unknown |
| SBU = Stony Brook University; NYSPI = New York State Psychiatric Institute; HCP = Human Connectome Project; ABCD = Adolescent Brain Cognitive Development; SD = Standard Deviation; M/F = Male/Female; W = White; AA = African American; AS = Asian; MR = Multiracial; H = Hispanic; NH = Non-Hispanic  **Note:** for three ABCD participants, only information on sex was available and was used in place of gender. Race/ethnicity information was missing for these participants as well, and they are reported “Unknown.” | | | | | |

**Table S1.** Demographic information for all datasets used in this study.

| Table S2. Single-band fMRI datasets used in this study. | | | | | | | | | | | |
| --- | --- | --- | --- | --- | --- | --- | --- | --- | --- | --- | --- |
| Dataset ID | **Scanner(s)** | **# Subjects** | **Phantom/In Vivo** | **TR (ms)** | **TE (ms)** | **FOV (mm)** | **Matrix Size** | **SENSE Factor** | **Flip Angle (°)** | **Voxel Size** | **Task(s)** |
| NYSPI-Philips | Philips Intera 1.5T | N = 27 | In Vivo | 2000 | 28 | 192x192 | 64x64 | 1.5 | 77 | 3 mm isotropic | SOT (N = 27), N-Back (N = 22) |
| QTIM-Bruker | Bruker MedSpec 4T | N = 46 | In Vivo | 2100 | 30 | 230x230 | 64x64 | N/A | 90 | 3.6x3.6x3.0 mm | RS (N = 46),  N-Back (N = 46) |
| NYSPI = New York State Psychiatric Institute; QTIM = Queensland Twin Imaging Study; TR = Repetition Time; TE = Echo Time; FOV = Field of View; SENSE = Sensitivity Encoding; RS = Resting-State; SOT = Self-Ordered Working Memory Task | | | | | | | | | | | |

**Table S3**. Mean Pearson correlations between simultaneously acquired (SA+) slices and adjacent-to-simultaneous (Adj) slices in uncorrected and MARSS-corrected data for all resting-state and task-based datasets.

|  | **Uncorrected Data** | | | **MARSS-Corrected Data** | | |
| --- | --- | --- | --- | --- | --- | --- |
| **Dataset** | $\boldsymbol{r}_{\boldsymbol{SA+}}$ | $\boldsymbol{r}_{\boldsymbol{Adj}}$ | $\boldsymbol{\Delta}\boldsymbol{r}_{\boldsymbol{(SA+ - Adj)}}$  $\boldsymbol{(}\boldsymbol{\Delta}\boldsymbol{Z)}$ | $\boldsymbol{r}_{\boldsymbol{SA+}}$ | $\boldsymbol{r}_{\boldsymbol{Adj}}$ | $\boldsymbol{\Delta}\boldsymbol{r}_{\boldsymbol{(SA+ - Adj)}}$  $\boldsymbol{(}\boldsymbol{\Delta}\boldsymbol{Z)}$ |
| NYSPI-GE RS | 0.608 | 0.228 | 0.380 (0.400) | 0.379 | 0.447 | -0.068 (-0.068) |
| NYSPI-GE SOT | 0.604 | 0.146 | 0.458 (0.494) | 0.293 | 0.378 | -0.085 (-0.085) |
| SBU-Siemens RS | 0.682 | 0.283 | 0.399 (0.423) | 0.395 | 0.433 | -0.038 (-0.038) |
| SBU-Siemens SOT | 0.667 | 0.234 | 0.433 (0.464) | 0.256 | 0.299 | -0.043 (-0.043) |
| HCP-Siemens RS | 0.631 | 0.217 | 0.414 (0.440) | 0.429 | 0.465 | -0.036 (-0.036) |
| HCP-Siemens WM | 0.574 | 0.109 | 0.465 (0.504) | 0.261 | 0.311 | -0.050 (-0.050) |
| ABCD-Siemens RS | 0.870 | 0.600 | 0.270 (0.276) | 0.836 | 0.861 | -0.025 (-0.025) |
| ABCD-GE RS | 0.667 | 0.438 | 0.229 (0.233) | 0.599 | 0.578 | 0.021 (0.021) |
| ABCD-Philips RS | 0.540 | 0.168 | 0.372 (0.391) | 0.288 | 0.035 | -0.062 (-0.062) |
| RS = Resting-State; SOT = Self-Ordered Working Memory Task; WM = Working Memory Task; $r_{SA+}$= Mean Pearson correlation between simultaneously acquired slices; $r_{Adj}$= Mean Pearson correlation between adjacent slices; $\Delta r_{(SA+ - Adj)}$ = Mean difference in Pearson correlation between simultaneously acquired slices and adjacent slices; $\boldsymbol{\Delta}\boldsymbol{Z}$ = Mean difference in Z-transformed Pearson correlation between simultaneously acquired slices and adjacent slices. | | | | | | |

**Table S4.** Mean Pearson correlations between simultaneously acquired (SA+) slices and adjacent-to-simultaneous (Adj) slices in phantom and in vivo resting-state data with varying multiband (MB) factors in uncorrected and MARSS-corrected data.

|  |  |  | Uncorrected Data | | | MARSS-Corrected Data | | |
| --- | --- | --- | --- | --- | --- | --- | --- | --- |
| Dataset | **Phantom/In Vivo** | **MB Factor** | $\boldsymbol{r}_{\boldsymbol{SA+}}$ | $\boldsymbol{r}_{\boldsymbol{Adj}}$ | $\boldsymbol{\Delta}\boldsymbol{r}_{\left( \boldsymbol{SA+ - Adj} \right)}$  $\boldsymbol{(}\boldsymbol{\Delta}\boldsymbol{Z)}$ | $\boldsymbol{r}_{\boldsymbol{SA+}}$ | $\boldsymbol{r}_{\boldsymbol{Adj}}$ | $\boldsymbol{\Delta}\boldsymbol{r}_{\boldsymbol{(SA+ - Adj)}}$  $\boldsymbol{(}\boldsymbol{\Delta}\boldsymbol{Z}\boldsymbol{)}$ |
| NYSPI-GE | In Vivo | 3  5  7 | 0.539 | 0.249 | 0.290 (0.298) | 0.245 | 0.368 | -0.123 (-0.124) |
|  |  |  | 0.569 | 0.188 | 0.364 (0.381) | 0.324 | 0.428 | -0.104 (-0.104) |
|  |  |  | 0.592 | 0.335 | 0.257 (0.263) | 0.467 | 0.482 | -0.015 (-0.015) |
|  | Phantom | 4  6  8 | 0.214 | 0.009 | 0.205 (0.208) | 0.021 | 0.067 | -0.046 (-0.046) |
|  |  |  | 0.226 | 0.106 | 0.120 (0.121) | 0.037 | 0.106 | -0.069 (-0.069) |
|  |  |  | 0.215 | 0.068 | 0.147 (0.148) | -0.094 | -0.004 | -0.090 (-0.090) |
| SBU-Siemens | In Vivo | 3  5  7 | 0.689 | 0.448 | 0.241 (0.246) | 0.501 | 0.523 | -0.022 (-0.022) |
|  |  |  | 0.624 | 0.225 | 0.399 (0.422) | 0.321 | 0.334 | -0.013 (-0.013) |
|  |  |  | 0.692 | 0.282 | 0.410 (0.436) | 0.435 | 0.459 | -0.024 (-0.024) |
|  | Phantom | 4  6  8 | 0.212 | 0.007 | 0.205 (0.208) | -0.112 | 0.035 | -0.147 (-0.148) |
|  |  |  | 0.213 | 0.057 | 0.156 (0.158) | -0.056 | 0.049 | -0.105 (-0.106) |
|  |  |  | 0.189 | 0.044 | 0.145 (0.146) | -0.020 | 0.052 | -0.072 (-0.072) |
| $\boldsymbol{r}_{\boldsymbol{SA+}}$= Mean Pearson correlation between simultaneously acquired slices; $\boldsymbol{r}_{\boldsymbol{Adj}}$= Mean Pearson correlation between adjacent slices; $\boldsymbol{\Delta}\boldsymbol{r}_{\boldsymbol{(SA+ - Adj)}}$ = Mean difference in Pearson correlation between simultaneously acquired slices and adjacent slices; $\boldsymbol{\Delta}\boldsymbol{Z}$ = Mean difference in Z-transformed Pearson correlation between simultaneously acquired slices and adjacent slices. | | | | | | | | |

**Table S5**. Results of Wilcoxon Signed Rank tests to determine the significance of the reduction in correlation difference between simultaneously acquired slices and adjacent slices following MARSS artifact correction.

| **Dataset** | **n** | **W** | **p** |
| --- | --- | --- | --- |
| NYSPI-GE RS | 232 | 27,028 | 8.21 x 10^-40^ |
| NYSPI-GE SOT | 186 | 17,578 | 1.94 x 10^-32^ |
| SBU-Siemens RS | 42 | 903 | 1.65 x 10^-8^ |
| SBU-Siemens SOT | 39 | 780 | 5.26 x 10^-8^ |
| HCP-Siemens RS | 100 | 5,050 | 3.90 x 10^-18^ |
| HCP-Siemens WM | 50 | 1,275 | 7.56 x 10^-10^ |
| ABCD-Siemens RS | 81 | 3,321 | 5.36 x 10^-15^ |
| ABCD-GE RS | 9 | 45 | 0.0039 |
| ABCD-Philips RS | 21 | 231 | 5.96 x 10^-5^ |
| RS = Resting-State; SOT = Self-Ordered Working Memory Task; WM = Working Memory Task; n = number of runs in each dataset; W = Wilcoxon signed rank test statistic; p = p-value | | | |

| **Table S6.** Mean Pearson correlations between simultaneously acquired (SA+) slices and adjacent-to-simultaneous (Adj) slices in NYSPI-GE task-based data (N = 58) that has been corrected by standard MARSS procedures, as well as MARSS procedures with artifact signal intensity thresholds (MARSS30, MARSS20, and MARSS10). | | | |
| --- | --- | --- | --- |
|  | **MARSS-Corrected Data** | | |
| **Dataset** | $\boldsymbol{r}_{\boldsymbol{SA+}}$ | $\boldsymbol{r}_{\boldsymbol{Adj}}$ | $\boldsymbol{\Delta}\boldsymbol{r}_{\boldsymbol{(SA+ - Adj)}}$  $\boldsymbol{(}\boldsymbol{\Delta}\boldsymbol{Z)}$ |
| NYSPI-GE SOT MARSS | 0.293 | 0.378 | -0.085 (-0.085) |
| NYSPI-GE SOT MARSS30 | 0.341 | 0.365 | -0.024 (-0.024) |
| NYSPI-GE SOT MARSS20 | 0.369 | 0.353 | 0.016 (0.016) |
| NYSPI-GE SOT MARSS10 | 0.413 | 0.327 | 0.086 (0.086) |
| SOT = Self-Ordered Working Memory Task; $r_{SA+}$= Mean Pearson correlation between simultaneously acquired slices; $r_{Adj}$= Mean Pearson correlation between adjacent slices; $\Delta r_{(SA+ - Adj)}$ = Mean difference in Pearson correlation between simultaneously acquired slices and adjacent slices; $\boldsymbol{\Delta}\boldsymbol{Z}$ = Mean difference in Z-transformed Pearson correlation between simultaneously acquired slices and adjacent slices. | | | |

| **Dataset** | $\boldsymbol{tSN}\boldsymbol{R}_{\boldsymbol{uncorrected}}$ | $\boldsymbol{tSN}\boldsymbol{R}_{\boldsymbol{MARSS}}$ | $\boldsymbol{\Delta}\boldsymbol{tSNR}$ | **n** | **W** | **p** |
| --- | --- | --- | --- | --- | --- | --- |
| NYSPI-GE RS | 18.970 | 19.027 | 0.057 | 232 | 27,028 | 8.201 x 10^-40^ |
| NYSPI-GE SOT | 17.649 | 17.705 | 0.056 | 186 | 17,578 | 1.944 x 10^-32^ |
| SBU-Siemens RS | 29.161 | 29.363 | 0.202 | 42 | 903 | 1.648 x 10^-8^ |
| SBU-Siemens SOT | 27.192 | 27.416 | 0.224 | 39 | 780 | 5.255 x 10^-8^ |
| HCP-Siemens RS | 16.614 | 16.665 | 0.051 | 100 | 5,050 | 3.897 x 10^-18^ |
| HCP-Siemens WM | 19.456 | 19.535 | 0.079 | 50 | 1,275 | 7.557 x 10^-10^ |
| ABCD-Siemens RS | 23.921 | 24.090 | 0.169 | 81 | 3,321 | 5.363 x 10^-15^ |
| ABCD-GE RS | 25.336 | 25.210 | 0.126 | 9 | 45 | 0.004 |
| ABCD-Philips RS | 23.822 | 24.043 | 0.221 | 21 | 231 | 5.957 x 10^-5^ |
| RS = Resting-State; SOT = Self-Ordered Working Memory Task; WM = Working Memory Task; $tSNR$ = temporal signal-to-noise ratio; n = number of runs in each dataset; W = Wilcoxon Signed Rank test statistic; p = p-value. | | | | | | |

**Table S7.** Change in temporal signal-to-noise ratio (tSNR) as a result of MARSS artifact correction in all unprocessed, resting-state and task-based fMRI datasets. Elevations in tSNR were tested for statistical significance via Wilcoxon Signed Rank tests.

| **Table S8.** Mean Pearson correlations between simultaneously acquired (SA+) slices and adjacent-to-simultaneous (Adj) slices in NYSPI-GE resting-state (N = 63) and task-based (N = 58) data, calculated using only voxels containing gray matter, white matter, and cerebrospinal fluid, respectively. | | | | | | |
| --- | --- | --- | --- | --- | --- | --- |
|  | **Uncorrected Data** | | | **MARSS-Corrected Data** | | |
| **Dataset** | $\boldsymbol{r}_{\boldsymbol{SA+}}$ | $\boldsymbol{r}_{\boldsymbol{Adj}}$ | $\boldsymbol{\Delta}\boldsymbol{r}_{\boldsymbol{(SA+ - Adj)}}$  $\boldsymbol{(}\boldsymbol{\Delta}\boldsymbol{Z)}$ | $\boldsymbol{r}_{\boldsymbol{SA+}}$ | $\boldsymbol{r}_{\boldsymbol{Adj}}$ | $\boldsymbol{\Delta}\boldsymbol{r}_{\boldsymbol{(SA+ - Adj)}}$  $\boldsymbol{(}\boldsymbol{\Delta}\boldsymbol{Z)}$ |
| NYSPI-GE RS Gray Matter | 0.638 | 0.311 | 0.327 (0.339) | 0.525 | 0.471 | 0.054 (0.054) |
| NYSPI-GE RS White Matter | 0.442 | 0.106 | 0.336 (0.350) | 0.292 | 0.170 | 0.122 (0.122) |
| NYSPI-GE RS CSF | 0.109 | -0.003 | 0.112 (0.112) | 0.070 | 0.015 | 0.055 (0.055) |
| NYSPI-GE SOT Gray Matter | 0.617 | 0.200 | 0.417 (0.444) | 0.438 | 0.387 | 0.051 (0.051) |
| NYSPI-GE SOT White Matter | 0.493 | 0.043 | 0.450 (0.485) | 0.271 | 0.134 | 0.137 (0.138) |
| NYSPI-GE SOT CSF | 0.120 | -0.015 | 0.135 (0.136) | 0.065 | 0.008 | 0.057 (0.057) |
| RS = Resting-State; SOT = Self-ordered Working Memory Task; CSF = Cerebrospinal Fluid $r_{SA+}$= Mean Pearson correlation between simultaneously acquired slices; $r_{Adj}$= Mean Pearson correlation between adjacent slices; $\Delta r_{(SA+ - Adj)}$ = Mean difference in Pearson correlation between simultaneously acquired slices and adjacent slices; $\boldsymbol{\Delta}\boldsymbol{Z}$ = Mean difference in Z-transformed Pearson correlation between simultaneously acquired slices and adjacent slices. | | | | | | |

| **Table S9.** Results of Wilcoxon Signed Rank tests to determine the significance of the reduction in correlation difference between simultaneously acquired slices and adjacent slices in gray matter, white matter, and cerebrospinal fluid following MARSS correction. | | | |
| --- | --- | --- | --- |
| **Dataset** | **n** | **W** | **p** |
| NYSPI-GE RS Gray Matter | 232 | 27,028 | 8.201 x 10^-40^ |
| NYSPI-GE RS White Matter | 232 | 27,028 | 8.201 x 10^-40^ |
| NYSPI-GE RS CSF | 232 | 26,849 | 8.242 x 10^-39^ |
| NYSPI-GE SOT Gray Matter | 186 | 17,578 | 1.944 x 10^-32^ |
| NYSPI-GE SOT White Matter | 186 | 17,578 | 1.944 x 10^-32^ |
| NYSPI-GE SOT CSF | 186 | 17,566 | 2.359 x 10^-32^ |
| RS = Resting-State; SOT = Self-ordered Working Memory Task; CSF = Cerebrospinal Fluid; n = number of runs; W = Wilcoxon signed rank test statistic; p = p-value | | | |

| **Table S10.** Change in temporal signal-to-noise ratio (tSNR) as a result of MARSS correction in unprocessed, NYSPI-GE resting-state (N = 63) and task-based (N = 58) datasets, calculated separately for gray matter, white matter, and cerebrospinal fluid. Elevations in tSNR were tested for statistical significance via Wilcoxon Signed Rank tests. | | | | | | | |
| --- | --- | --- | --- | --- | --- | --- | --- |
| **Dataset** | $\boldsymbol{tSN}\boldsymbol{R}_{\boldsymbol{uncorrected}}$ | $\boldsymbol{tSN}\boldsymbol{R}_{\boldsymbol{MARSS}}$ | $\boldsymbol{\Delta}\boldsymbol{tSNR}$ | **n** | **W** | **p** | **Artifact Magnitude (σ)** |
| NYSPI-GE RS Gray Matter | 22.897 | 22.972 | 0.075 | 232 | 27,028 | 8.201 x 10^-40^ | 0.505 (0.173) |
| NYSPI-GE RS White Matter | 24.950 | 25.002 | 0.052 | 232 | 27,028 | 8.201 x 10^-40^ | 0.336 (0.112) |
| NYSPI-GE RS CSF | 17.268 | 17.346 | 0.078 | 232 | 27,028 | 8.201 x 10^-40^ | 0.710 (0.326) |
| NYSPI-GE SOT Gray Matter | 21.103 | 21.179 | 0.076 | 186 | 17,578 | 1.944 x 10^-32^ | 0.575 (0.230) |
| NYSPI-GE SOT White Matter | 23.475 | 23.534 | 0.059 | 186 | 17,578 | 1.944 x 10^-32^ | 0.389 (0.155) |
| NYSPI-GE SOT CSF | 16.473 | 16.552 | 0.079 | 186 | 17,578 | 1.944 x 10^-32^ | 0.701 (0.305) |
| RS = Resting-State; SOT = Self-ordered Working Memory Task; CSF = Cerebrospinal Fluid; $tSNR$ = temporal signal-to-noise ratio; n = number of runs in each dataset; W = Wilcoxon Signed Rank test statistic; p = p-value. | | | | | | | |

| **Table S11.** Mean Pearson correlations between simultaneously acquired (SA+) slices and adjacent-to-simultaneous (Adj) slices in uncorrected HCP-Siemens resting-state data (N = 25), as well as data that has undergone physiological correction via RETROICOR alone, MARSS correction alone and MARSS prior to RETROICOR. | | | |
| --- | --- | --- | --- |
| **Dataset** | $\boldsymbol{r}_{\boldsymbol{SA+}}$ | $\boldsymbol{r}_{\boldsymbol{Adj}}$ | $\boldsymbol{\Delta}\boldsymbol{r}_{\boldsymbol{(SA+ - Adj)}}$  $\boldsymbol{(}\boldsymbol{\Delta}\boldsymbol{Z)}$ |
| HCP-Siemens RS Uncorrected | 0.631 | 0.217 | 0.414 (0.440) |
| HCP-Siemens RS RETROICOR-Corrected | 0.534 | 0.312 | 0.222 (0.226) |
| HCP-Siemens RS MARSS-Corrected | 0.429 | 0.465 | -0.036 (-0.036) |
| HCP-Siemens RS MARSS+RETROICOR-Corrected | 0.377 | 0.388 | -0.011 (-0.011) |
| RS = Resting-State; $r_{SA+}$= Mean Pearson correlation between simultaneously acquired slices; $r_{Adj}$= Mean Pearson correlation between adjacent slices; $\Delta r_{(SA+ - Adj)}$ = Mean difference in Pearson correlation between simultaneously acquired slices and adjacent slices; $\boldsymbol{\Delta}\boldsymbol{Z}$ = Mean difference in Z-transformed Pearson correlation between simultaneously acquired slices and adjacent slices. | | | |

**Table S12**. Mean Pearson correlations between simultaneously acquired (SA+) slices and adjacent-to-simultaneous (Adj) slices in subspaces of sICA+FIX denoised, resting-state data from the HCP-Siemens dataset.

|  | **Uncorrected Data** | | | **MARSS-Corrected Data** | | | **Pre-MARSS ̶ Post-MARSS** | | |
| --- | --- | --- | --- | --- | --- | --- | --- | --- | --- |
| **Data** | $\boldsymbol{r}_{\boldsymbol{SA+}}$ | $\boldsymbol{r}_{\boldsymbol{Adj}}$ | $\boldsymbol{\Delta}\boldsymbol{r}_{\boldsymbol{(SA+ - Adj)}}$  $\boldsymbol{(}\boldsymbol{\Delta}\boldsymbol{Z)}$ | $\boldsymbol{r}_{\boldsymbol{SA+}}$ | $\boldsymbol{r}_{\boldsymbol{Adj}}$ | $\boldsymbol{\Delta}\boldsymbol{r}_{\boldsymbol{(SA+ - Adj)}}$  $\boldsymbol{(}\boldsymbol{\Delta}\boldsymbol{Z)}$ | $\boldsymbol{r}_{\boldsymbol{SA+}}$ | $\boldsymbol{r}_{\boldsymbol{Adj}}$ | $\boldsymbol{\Delta}\boldsymbol{r}_{\boldsymbol{(SA+ - Adj)}}$  $\boldsymbol{(}\boldsymbol{\Delta}\boldsymbol{Z)}$ |
| sICA+FIX Cleaned | 0.517 | 0.341 | 0.176  (0.178) | 0.396 | 0.398 | -0.002  (-0.002) | 0.960 | -0.017 | 0.977  (2.232) |
| Neural Signal Subspace | 0.707 | 0.626 | 0.081  (0.081) | 0.622 | 0.624 | -0.002  (-0.002) | 0.932 | -0.016 | 0.948  (1.806) |
| Random Noise Subspace | 0.275 | -0.020 | 0.295  (0.304) | 0.052 | 0.053 | -0.001  (-0.001) | 0.967 | -0.016 | 0.983  (2.382) |
| sICA+FIX Noise Components | 0.634 | 0.120 | 0.514  (0.568) | 0.450 | 0.428 | 0.022  (0.022) | 0.989 | -0.007 | 0.996  (3.061) |
| Neural Subspace + GSR | 0.052 | -0.109 | 0.161  (0.162) | -0.080 | -0.076 | -0.004  (-0.004) | 0.914 | -0.029 | 0.943  (1.767) |
| $r_{SA+}$= Mean Pearson correlation between simultaneously acquired slices; $r_{Adj}$= Mean Pearson correlation between adjacent slices; $\Delta r_{(SA+ - Adj)}$ = Mean difference in Pearson correlation between simultaneously acquired slices and adjacent slices; $\boldsymbol{\Delta}\boldsymbol{Z}$ = Mean difference in Z-transformed Pearson correlation between simultaneously acquired slices and adjacent slices. | | | | | | | | | |

|  | **Subspace Variance (Mean ± 2*Standard Error)** | | | | | |
| --- | --- | --- | --- | --- | --- | --- |
|  | $Var_{Detrend}$ | $Var_{MP24}$ | $Var_{NoiseICA}$ | $Var_{Neural}$ | $Var_{Random}$ | $Var_{MARSS}$ |
| **Uncorrected Data** | 124,206.114 ± 2,857.174 | 96,332.605 ± 1,674.362 | 27,583.954 ± 359.005 | 6,006.277 ± 80.249 | 61,903.802  ± 638.074 | N/A |
| **MARSS-Corrected Data** | 124,186.625 ± 2,857.116 | 96,323.032 ± 1,674.039 | 25,968.731 ± 349.502 | 6,016.725 ± 80.099 | 62,077.783  ± 640.134 | 1,522.526 ± 22.692 |
| $Var_{Detrend}$ = Variance calculated on signal removed via linear detrending; $Var_{MP24}$ = Variance calculated on signal removed via regression of 24 motion parameters; $Var_{NoiseICA}$ = Variance calculated on sICA+FIX noise components; $Var_{Neural}$ = Variance of the neural signal subspace; $Var_{Random}$ = Variance of the random noise subspace; $Var_{MARSS}$ = Variance of the signal removed via MARSS correction on raw, unprocessed data. | | | | | | |

**Table S13**. Decomposition of mean voxel-wise variance across participants in data subspaces, performed in both uncorrected and MARSS corrected resting-state data in the HCP-Siemens dataset.

**Supplementary References**

Friston, K. J. (2007). *Statistical parametric mapping : the analysis of funtional brain images* (1st ed.). Amsterdam ; Boston: Elsevier/Academic Press.

Friston, K. J., Holmes, A. P., Worsley, K. J., Poline, J. P., Frith, C. D., & Frackowiak, R. S. J. (2004). Statistical parametric maps in functional imaging: A general linear approach. *Human Brain Mapping, 2*(4), 189-210. doi:10.1002/hbm.460020402

Glasser, M. F., Coalson, T. S., Bijsterbosch, J. D., Harrison, S. J., Harms, M. P., Anticevic, A., . . . Smith, S. M. (2018). Using temporal ICA to selectively remove global noise while preserving global signal in functional MRI data. *Neuroimage, 181*, 692-717. doi:10.1016/j.neuroimage.2018.04.076

Marcus, D. S., Harwell, J., Olsen, T., Hodge, M., Glasser, M. F., Prior, F., . . . Van Essen, D. C. (2011). Informatics and data mining tools and strategies for the human connectome project. *Front Neuroinform, 5*, 4. doi:10.3389/fninf.2011.00004

Power, J. D., Barnes, K. A., Snyder, A. Z., Schlaggar, B. L., & Petersen, S. E. (2012). Spurious but systematic correlations in functional connectivity MRI networks arise from subject motion. *Neuroimage, 59*(3), 2142-2154. doi:10.1016/j.neuroimage.2011.10.018

Power, J. D., Mitra, A., Laumann, T. O., Snyder, A. Z., Schlaggar, B. L., & Petersen, S. E. (2014). Methods to detect, characterize, and remove motion artifact in resting state fMRI. *NeuroImage, 84*, 320-341. doi:10.1016/j.neuroimage.2013.08.048

Smyser, C. D., Inder, T. E., Shimony, J. S., Hill, J. E., Degnan, A. J., Snyder, A. Z., & Neil, J. J. (2010). Longitudinal analysis of neural network development in preterm infants. *Cereb Cortex, 20*(12), 2852-2862. doi:10.1093/cercor/bhq035

Van Essen, D. C., Curtiss, S. W., Laumann, T., Jenkinson, M., Prior, F., Glasser, M. F., . . . Marcus, D. S. (2011). Informatics and Data Mining Tools and Strategies for the Human Connectome Project. *Frontiers in Neuroinformatics, 5*. doi:10.3389/fninf.2011.00004

Van Snellenberg, J. X., Conway, A. R., Spicer, J., Read, C., & Smith, E. E. (2014). Capacity estimates in working memory: Reliability and interrelationships among tasks. *Cogn Affect Behav Neurosci, 14*(1), 106-116. doi:10.3758/s13415-013-0235-x

Van Snellenberg, J. X., Girgis, R. R., Horga, G., van de Giessen, E., Slifstein, M., Ojeil, N., . . . Abi-Dargham, A. (2016). Mechanisms of Working Memory Impairment in Schizophrenia. *Biol Psychiatry, 80*(8), 617-626. doi:10.1016/j.biopsych.2016.02.017

Van Snellenberg, J. X., Slifstein, M., Read, C., Weber, J., Thompson, J. L., Wager, T. D., . . . Smith, E. E. (2015). Dynamic shifts in brain network activation during supracapacity working memory task performance. *Hum Brain Mapp, 36*(4), 1245-1264. doi:10.1002/hbm.22699

Welch, P. (1967). The use of fast Fourier transform for the estimation of power spectra: A method based on time averaging over short, modified periodograms. *IEEE Transactions on Audio and Electroacoustics, 15*(2), 70-73. doi:10.1109/tau.1967.1161901
